# Reduction of DNA Topoisomerase Top2 Reprograms the Epigenetic Landscape and Extends Health and Life Span Across Species

**DOI:** 10.1111/acel.70010

**Published:** 2025-02-12

**Authors:** Man Zhu, Meng Ma, Lunan Luo, Feiyang Li, Jiashun Zheng, Yan Pan, Lu Yang, Ying Xiao, Ziyan Wang, Bo Xian, Yi Zheng, Hao Li, Jing Yang

**Affiliations:** ^1^ Department of Health Management & Institute of Health Management, Sichuan Provincial People's Hospital University of Electronic Science and Technology of China Chengdu China; ^2^ Laboratory of Aging Research, School of Medicine University of Electronic Science and Technology of China Chengdu China; ^3^ Department of Biochemistry and Biophysics University of California San Francisco San Francisco California USA

**Keywords:** aging, essential gene, histone modification, longevity, topoisomerase II

## Abstract

DNA topoisomerases are essential molecular machines that manage DNA topology in the cell and play important roles in DNA replication and transcription. We found that knocking down the enzyme topoisomerase Top2 or its mammalian homolog TOP2B increases the lifespan of *S. cerevisiae* , *C. elegans* , and mice. TOP2B reduction also extends the health span of mice and alleviates the pathologies of aging in multiple tissues. At the cellular/molecular level, TOP2B reduction alleviates the major hallmarks of aging, including senescence, DNA damage, and deregulated nutrient sensing. We observed that TOP2B reduction changes the epigenetic landscape of various tissues in old mice toward that of the young animals, and differentially downregulates genes with active promoter and high expression. Our observations suggest that Top2 reduction confers pro‐longevity effect across species possibly through a conserved mechanism and may be a promising strategy for longevity intervention.

## Graphical abstract

This study demonstrates that reducing the enzyme topoisomerase Top2 or its mammalian homolog TOP2B promotes longevity across multiple species, including yeast, worms, and mice. At the cellular/molecular level, TOP2B reduction mitigates the major cellular aging hallmarks such as epigenetic alterations, cell senescence, lysosomal biogenesis, and deregulated nutrient sensing.

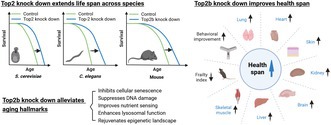

## 1 Introduction

The proportion of the global population aged 65 years and above is projected to increase from 10% in 2022 to 16% by 2050 (Wang and Wang 2021). The rapid increase in the elderly population has drawn worldwide interest in efforts to combat aging. Over the past few decades, the application of molecular genetics to model organisms has led to the discovery of several biological pathways that regulate lifespan across species, leading to insight into the mechanisms of aging and the development of potential therapeutic interventions (Guarente et al. 2024; López‐Otín et al. 2023). An important revelation from these studies is that some of the longevity pathways have essential cellular functions and are important for growth and development; however, their downregulation post‐development significantly extends lifespan. Examples include the classic insulin/IGF signaling and the TOR pathways (Kennedy and Lamming 2016; Kenyon 2010); their discovery led to the development of promising potential antiaging drugs currently under human clinical trial (Guarente et al. 2024).

To further our understanding of the mechanisms of aging and to identify alternative therapeutic targets, it is desirable to explore other key molecular machines/pathways for their role in regulating lifespan across species. However, information on the lifespan phenotype of essential genes is scarce, especially for mammalian species. In the simple model organism yeast, the lifespans of the nonessential gene knockout mutants have been measured systematically through a multi‐year effort and ~200 mutants with extended lifespans were identified (McCormick et al. 2015). As a significant fraction of the nonessential gene knockout mutants have been profiled transcriptionally (Kemmeren et al. 2014), we analyzed the correlation between the gene expression profile and the lifespan of the mutants and identified a number of essential genes whose downregulation strongly correlates with extended lifespan across multiple mutants. Among the top hits is the DNA topoisomerase Top2, with an essential function in managing DNA topology and regulating replication and transcription (Tammaro et al. 2013; Yan et al. 2016). Combined with the previous observation that reducing Top2 extends lifespan in yeast (Tombline et al. 2017), we hypothesized that Top2 serves as a key node in the gene regulatory networks that influence lifespan and that reducing Top2 might extend lifespan across species through a conserved mechanism.

Yeast Top2 has two mammalian homologs, TOP2A and TOP2B. While TOP2A is primarily expressed in proliferating cells and is crucial for DNA replication, TOP2B is expressed in all cell types and plays a more prominent role in DNA replication, chromatin remodeling, and transcriptional regulation that is closely tied to aging (Uusküla‐Reimand and Wilson 2022; Zhu et al. 2024). We thus decided to focus on TOP2B. TOP2B is an essential double‐stranded DNA topoisomerase, pivotal in identifying DNA topological configurations and relieving DNA torsional strain via cutting, rotating, and reconnecting DNA strands (Pommier et al. 2022). Emerging research underscores its importance in chromosome architecture maintenance, DNA replication and repair, and transcriptional regulation (Martínez‐García et al. 2021; Pommier et al. 2022). TOP2B has been much less studied in the context of aging, with a few previous studies implicating it in age‐related retinopathies and hearing impairment (Li et al. 2017; Xia et al. 2019), and downstream response to dietary restriction (Andrawus et al. 2020).

In this study, we investigate whether reduction of Top2 or TOP2B confers longevity phenotype across species and explore the potential mechanisms. We found that knocking down Top2 or TOP2B extends the lifespan of yeast, *C. elegans* , and mice. TOP2B reduction also extends the health span of mice, and alleviates the characteristics and pathologies of aging in multiple tissues. At the cellular/molecular level, Top2 or TOP2B reduction attenuates the major hallmarks of aging, such as cellular senescence, deregulated nutrient‐sensing, epigenetic alterations, and lysosomal dysfunction. We observed that TOP2B reduction alters the epigenetic landscape of various tissues in old mice toward those of the young animals, and differentially downregulates genes with active promoter and high expression. Our observations suggest that Top2 or TOP2B reduction confers longevity effect via remodeling of epigenetic and transcriptional landscapes and suppression of aberrantly expressed genes in old cells. Our findings also suggest TOP2B can be a promising therapeutic target for longevity intervention.

## 2 Results

### 2.1 Reduction of Top2 or Its Vertebrate Homolog TOP2B Extends the Lifespan of Yeast, Worms, and Mice

We begin with a bioinformatic analysis to identify essential genes whose perturbation may lead to lifespan extension in yeast. Using the systematic lifespan data from nonessential gene deletion mutants (McCormick et al. 2015) and the collection of gene expression profiles for a subset of the mutants (Kemmeren et al. 2014), we performed a regression analysis to identify essential genes whose expression change correlates with the lifespan change across different mutants (see Section 4). This analysis identified a number of essential genes with significant correlation, among them including Top2, a previous study found that knocking down Top2 extends the replicative lifespan of yeast (Tombline et al. 2017).

**FIGURE 1:**
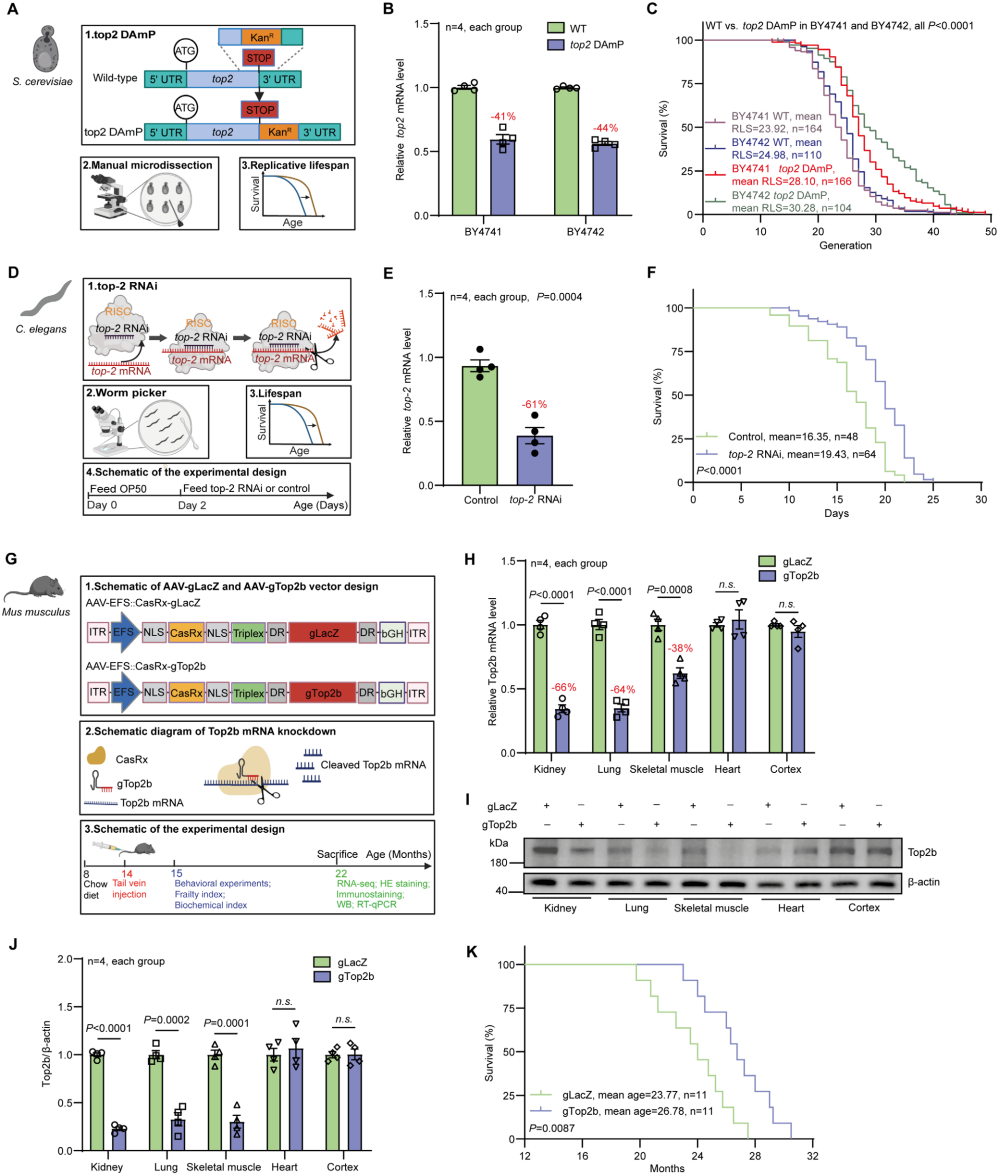
Reduction of Top2/TOP2B extends the life span of yeast, worm, and mice. (A) Schematic of Top2 DAmP approach in *S.cerevisiae*. DAmP, decreased abundance by mRNA perturbation; Kan^R^, kanamycin resistance. (B) Relative Top2 mRNA levels in BY4741 and BY4742 wild‐type (WT) strains and the corresponding Top2 DAmP mutants, as measured by RT‐qPCR. (C) Replicative life span (RLS) of BY4741 and BY4742 WT strains and the top2 DAmP mutants. (D) Schematic of *top‐2* RNAi approach in *C. elegans* . RNAi, RNA interference; RISC, RNA‐induced silencing complex. (E) Relative *top‐2* mRNA levels in *C. elegans* for the control group and the *top‐2* RNAi groups, as measured by RT‐qPCR. (F) Life span of the *C. elegans* for the control and *top‐2* RNAi groups. (G) Schematic of TOP2B knockdown by the CRISPR/CasRx system in mice. AAV, adeno‐associated virus. (H) Relative TOP2B mRNA levels in the kidneys, lungs, skeletal muscles, hearts, and cortices of mice in the gLacZ control and gTOP2B groups, measured by RT‐qPCR. (I–J) Relative TOP2B protein levels in the kidneys, lungs, skeletal muscles, hearts, and cortices of mice in the gLacZ control and gTOP2B groups, as measured by western blot. (K) The life span of C57BL/6 mice for the gLacZ control and gTOP2B groups. Statistical analysis was performed using GraphPad Prism v8.0 software (https://www.graphpad.com). Data were considered statistically significant at *p* < 0.05 calculated by using Student's *t*‐test (B, E, H, and J) or log‐rank test (C, F, and K). n.s. indicates not significant. All values are means ± SEM. The corresponding n values (number of cells, worms, or mice) are shown within each sub‐plot.

To investigate whether Top2 knockdown extends lifespan across species, we first test whether we can replicate the previously observed lifespan extension by Top2 knockdown in yeast (Tombline et al. 2017). We constructed Top2 DAmP (Decreased Abundance by mRNA Perturbation) strains in both the BY4741 (Mat‐a) and BY4742 (Mat‐alpha) genetic backgrounds. The DAmP strains have decreased abundance of the target mRNA because of 3′ modification that renders it less stable (Breslow et al. 2008). Compared to the wild‐type (WT) strains, the Top2 mRNA was knocked down by 41% and 44% in the TOP2 DAmP strains with BY4741 and BY4742 backgrounds respectively (Figure 1A,B). Compared to the BY4741 (mean RLS = 23.92, *n* = 164) and BY4742 WT (mean RLS = 24.98, *n* = 110) strains, the mean RLS of Top2 DAmP strains in BY4741 (mean RLS = 28.10, *n* = 166) and BY4742 (mean RLS = 30.28, *n* = 104) backgrounds was extended by 17.5% and 21.2%, respectively (Figure 1C, all *p* < 0.0001), as measured by using the traditional microdissection technique.

We next determined the effect of Top2 reduction on the lifespan of *C. elegans* using the N2 strain. We employed an RNAi approach (Figure 1D) to knock down the Top2 mRNA. With the RNAi approach, it is possible to analyze dose–response of the lifespan versus the degree of knockdown by feeding worms with different proportions of *E. coli* with the RNAi construct and the wild‐type *E. coli* (HT115 strain). We experimented with a range of different proportions of *E. coli* with Top2 RNAi (0%, 25%, 50%, 75%, 100%) and found lifespan extension in a dose‐dependent manner, with 50% yielding the largest lifespan extension (Extended Data Figure 1A,B). Compared to the control (mean lifespan = 16.35 days, *n* = 48), the lifespan of the 50% Top2 RNAi group (mean life span = 19.43 days, *n* = 64) was extended by 18.8% (Figure 1E,F, *p* < 0.0001); the mRNA expression level of Top2 was reduced to 61% of the WT level (Figure 1E). Interestingly, the optimal dose for the lifespan also yielded the best health indexes such as the number of pharyngeal beat per minute and percent of worms entered the slow swallowing phase (Extended Data Figure 1C–E). We therefore focused on 50% Top2 RNAi in the subsequent worm experiments.

Top2 is evolutionarily conserved in multicellular eukaryotes. TOP2A and TOP2B are two distinct forms in vertebrate species (Extended Data Figure 1F). TOP2A is primarily detected in actively dividing cells such as germ cells and is crucial for DNA replication. In contrast, TOP2B is widely and highly expressed in various tissues and cell types (Uusküla‐Reimand and Wilson 2022) and plays an important role in regulating chromatin structure and gene expression. To investigate whether Top2 knock down extends lifespan in mammals, we decided to focus on TOP2b and analyzed the effect on lifespan by TOP2b knock down in mice. We designed the CRISPR/CasRx system to knock down TOP2B and constructed AAV DJ serotype viruses to deliver the TOP2b knock down vector and the LacZ control into multiple organs/tissues through tail vein injection at 14 months age of the mice (Figure 1G). Real‐time quantitative PCR (RT‐qPCR) and Western blot analysis revealed that compared to the control group (gLacZ), the levels of TOP2B mRNA (Figure 1H) and protein (Figure 1I,J) in the kidneys, lungs, and skeletal muscles of TOP2B knockdown mice (gTOP2B group) were downregulated, despite no significant impact in the cortex and heart. In comparison to the gLacZ group (mean age = 23.77 months, *n* = 11), the average life span of gTOP2B group mice (mean age = 26.78 months, *n* = 11) was extended by 12.7% (Figure 1K; *p* = 0.0087).

### 2.2 TOP2B/Top2 Knockdown Improved the Health Span of Mice and *C. elegans*

**FIGURE 2:**
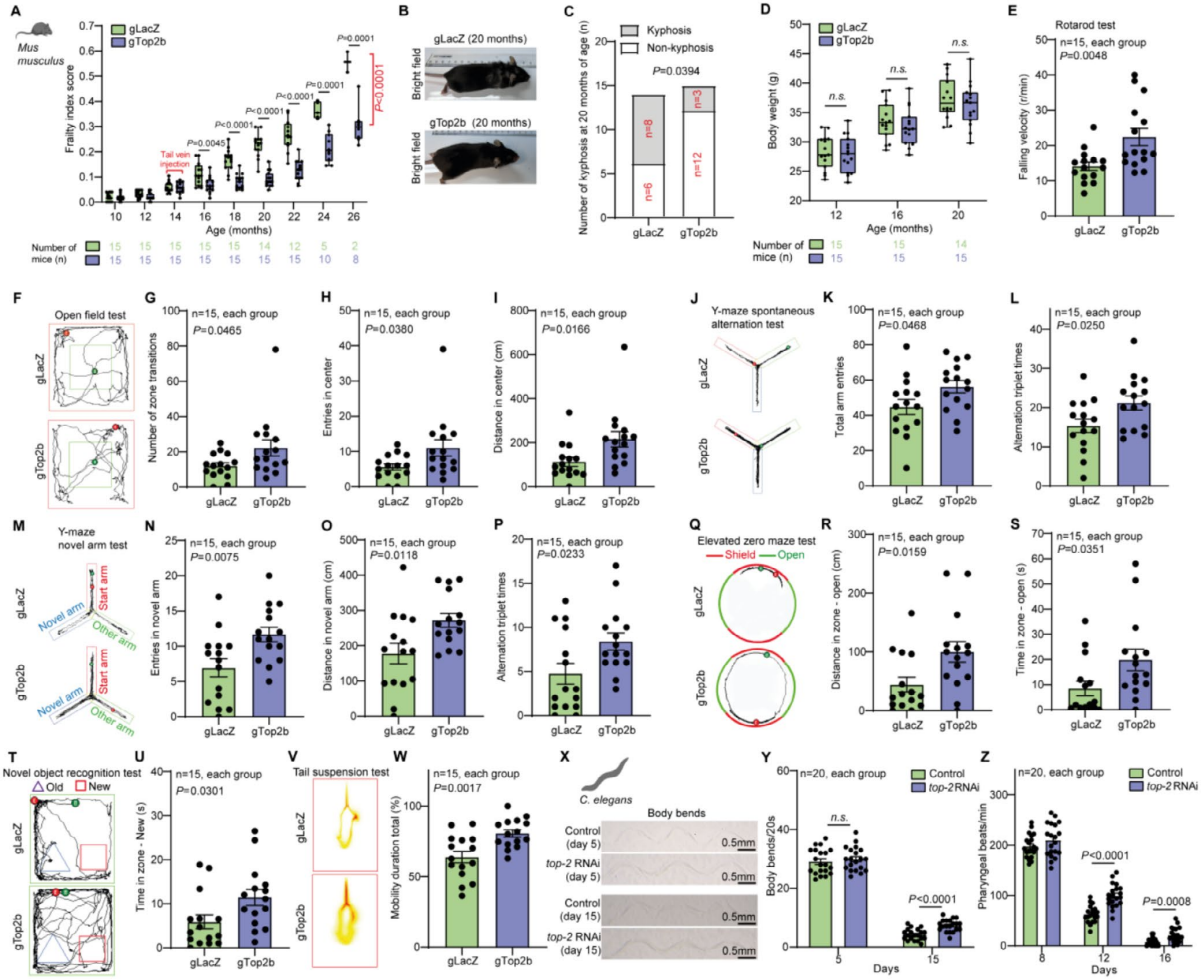
TOP2B/Top2 reduction improves the health span of mice and worms. (A) Frailty index (FI) scores for C57BL/6 mice in the gLacZ and gTOP2B groups were recorded at 10 to 26 months of age, with assessments conducted once every 2 months. The number of mice assessed at each age is listed at the bottom of the plot. (B) Bright‐field images of gLacZ and gTOP2B mice. (C) Number of kyphosis in C57BL/6 mice of the gLacZ and gTOP2B groups at 20 months of age. (D) Body weight of C57BL/6 mice in the gLacZ and gTOP2B groups at 12, 16, and 20 months of age. (E) Falling velocity in rotarod tests for C57BL/6 mice in the gLacZ and gTOP2B groups. (F–I) Representative images of open field tests and number of zone transitions, entries in the center, and distance in the center for C57BL/6 mice in the gLacZ and gTOP2B groups. (J–L) Representative images of Y‐maze spontaneous alternation tests and total arm entries and alternation triplet times for C57BL/6 mice in the gLacZ and gTOP2B groups. (M–P) Representative images of Y‐maze novel arm tests and entries in novel arm, distance in novel arm, and alternation triplet times for C57BL/6 mice in the gLacZ and gTOP2B groups. (Q–S) Representative images of elevated zero maze tests and distance in zone‐open and time in zone‐open for C57BL/6 mice in the gLacZ and gTOP2B groups. (T,U) Representative images of novel object recognition tests and time in zone‐new for C57BL/6 mice in the gLacZ and gTOP2B groups. (V, W) Representative images of tail suspension tests and mobility for C57BL/6 mice in the gLacZ and gTOP2B groups. (X–Z) Representative images of body bend and bending frequency and pharyngeal pumping rate in *C. elegans* fed with control RNAi and top‐2 RNAi after 5 and 15 days. Statistical analysis was performed using GraphPad Prism v8.0 software (https://www.graphpad.com). Data were considered statistically significant at *p* < 0.05 calculated by using Student's *t*‐test (D, E, G–I, K, L, N–P, R, S, U, W, Y, and Z) or Chi‐squared test (C) or two‐way ANOVA (A). n.s. indicates not significant. All values are means ± SEM or *n* (%). The corresponding *n* values (number of mice or worms) are shown in each sub‐plot.

An effective antiaging strategy should both extend life span and improve health span. The mouse frailty index (FI) is a comprehensive compilation of health indicators, encompassing body weight, coat condition, grip strength, mobility, vision, and hearing; lower FI indicates a more healthy condition (Feridooni et al. 2015). To assess the impact of TOP2B knockdown on the health span of mice, we conducted FI scoring every 2 months and performed extensive behavioral assays to evaluate the health status of the mice. No discernible differences in FI scores were observed between the two groups before AAV virus injection (at 14 months of age). However, FI scores of mice in the gTOP2B group were significantly lower than those in the gLacZ group after intravenous injection (Figure 2A, all *p* < 0.01).

At 20 months of age, a hair assessment was performed. We observed that mice in the gTOP2B group displayed a reduced incidence of alopecia/depigmentation phenotype and less pronounced spinal kyphosis compared to the gLacZ group (Figure 2B,C), and it seemed that TOP2B knockdown had a limited effect on the body weight (Figure 2D). Muscle coordination, endurance, and strength decline with age (Clayton et al. 2022). In the rotarod test, mice in the gTOP2B group showed superior performance at both latencies to fall and falling velocity compared to the gLacZ group (Figure 2E, Extended Data Figure 2A). Reduced memory and exploration and increased anxiety are common behavioral changes induced by aging (Wang et al. 2022). We conducted extensive behavioral experiments including the open field test (Figure 2F–I, Extended Data Figure 2B–D), Y‐maze spontaneous alternation test (Figure 2J–L, Extended Data Figure 2E,F), Y‐maze novel arm test (Figure 2M–P, Extended Data Figure 2G), elevated zero maze test (Figure 2Q–S, Extended Data Figure 2H–J), and novel object recognition test (Figure 2T,U, Extended Data Figure 2K). The open field test, Y‐maze spontaneous alteration test, and novel arm test assess different behavioral and cognitive functions, providing complementary insights into the etiology of aging. The open field test evaluates general locomotor activity and anxiety, while the Y‐maze spontaneous alteration test measures spatial working memory, sensitive to age‐related cognitive decline. The novel arm test assesses recognition memory and novelty processing, often impaired with aging (Piantadosi and Holmes 2023). Together, these tests offer a comprehensive understanding of the cognitive and behavioral changes associated with aging, highlighting specific areas affected by neurodegenerative processes. In this study, we found that compared to the gLacZ group, mice in the gTOP2B group demonstrated significantly enhanced spatial exploration and memory and exhibited lesser anxiety. Although depressive‐like behavior is not a typical manifestation of aging, aging undoubtedly serves as a crucial precipitant (Lorenzo et al. 2023). The tail suspension test also revealed that mice in the gTOP2B group exhibited less depressive‐like behavior compared to the gLacZ group (Figure 2V,W).

We also evaluated the benefit of Top2 knockdown to the health span of *C. elegans* . Bending frequency and pharyngeal pumping rate represent two pivotal metrics for evaluating age‐associated physiological deterioration in *C. elegans* , both exhibiting a progressive decline with advancing age. We found a significant increase in both bending frequency and pharyngeal pumping rate of *C. elegans* in the Top2 RNAi group compared to the HT115 group (Figure 2X–Z).

### 2.3 TOP2B Reduction Mitigates the Characteristics and Pathologies of Aging in Multiple Tissues in Mice

**FIGURE 3:**
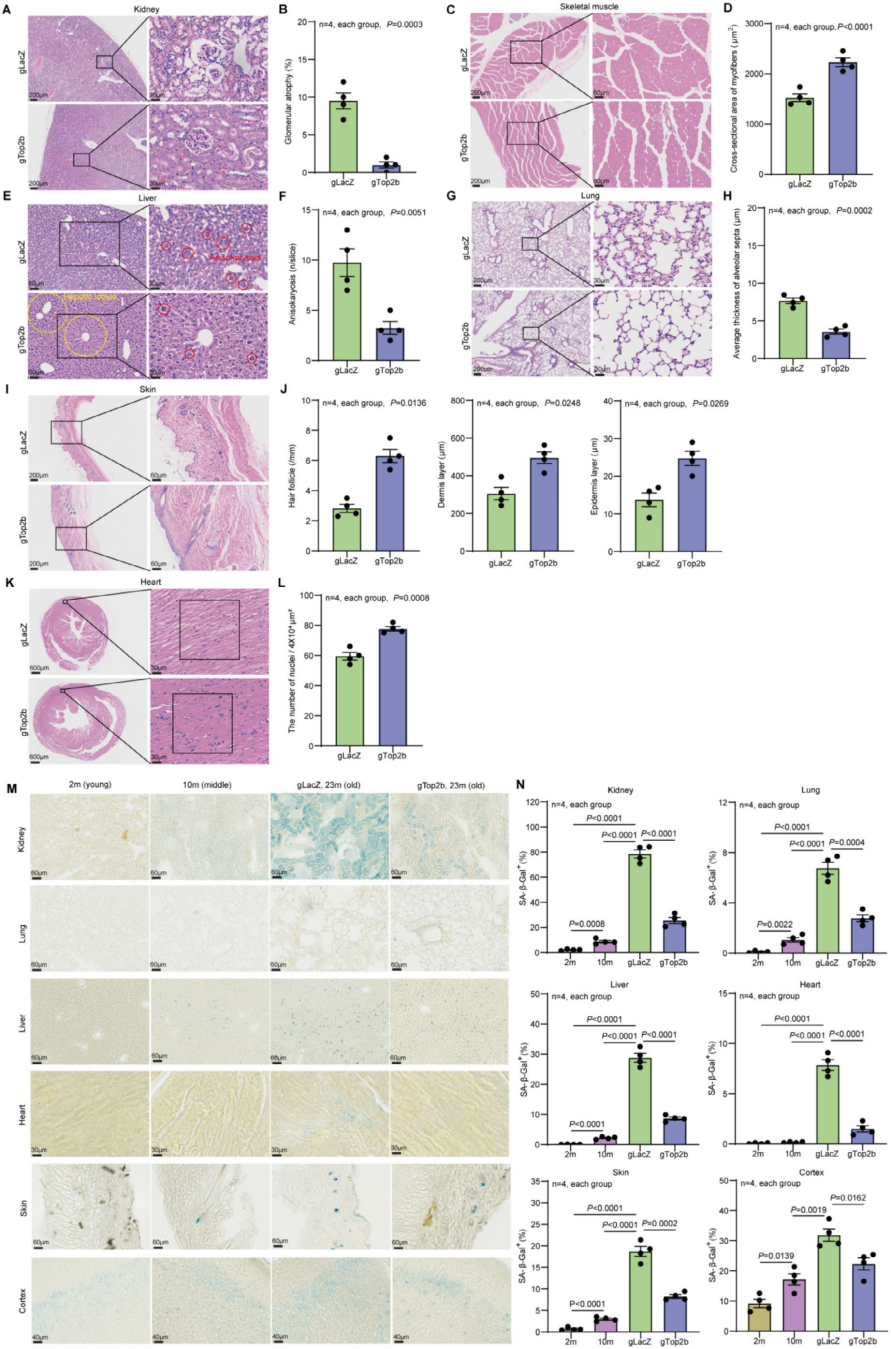
TOP2B reduction mitigates the characteristics and pathologies associated with aging in multiple mouse tissues. Histology of various tissues of 23‐month‐old mice in the gLacZ control group and gTop2 group is analyzed (A–L). (A) The longitudinal sections of the kidney tissues were stained with HE. The right panels show a magnified view of the boxed area in the left panels. Scale bars represent 200 and 30 μm for the left and right panels. (B) Average glomerular atrophy. (C) The transverse sections of mouse skeletal muscle were stained with HE. The right panels show a magnified view of the boxed area in the left panels. Scale bars represent 200 μm for the left panels and 60 μm for the right panels. (D) Average cross‐sectional area of myofibers. (E) Mouse liver tissues were stained with HE. The right panels show a magnified view of the boxed area in the left panels. Scale bars represent 60 μm for the left panels and 30 μm for the right panels. The yellow circles highlight the hepatic lobule. The red circles indicate anisokaryosis. (F) Average anisokaryosis. (G) Mouse lung tissues were stained with HE. The right panels show a magnified view of the boxed area in the left panels. Scale bars represent 200 μm for the left panels and 30 μm for the right panels. (H) Average thickness of alveolar septa. (I) Mouse skin tissues were stained with HE. The right panels show a magnified view of the boxed area in the left panels. Scale bars represent 200 μm for the left panels and 60 μm for the right panels. (J) Average hair follicle density, average dermis layer thickness, and average epidermis layer thickness in mouse skin. (K) The short axes of cardiac tissues from mice were stained with HE in the left ventricular wall regions. The right panels show a magnified view of the squared area in the left panels. Scale bars represent 600 and 30 μm for the left and right panels. (L) The number of nuclei in each region of the left ventricular wall. (M) Representative images of SA‐β‐Gal staining of various tissues from young (2 months old), middle age (10 months old), old (23 months old) gLacZ control, and old (23 months old) gTOP2B groups. (N) Quantitation of percent of SA‐β‐Gal positive cells in various tissues. Statistical analysis was performed using GraphPad Prism v8.0 software (https://www.graphpad.com). Data were considered statistically significant at *p* < 0.05 calculated by using Student's *t*‐test (B, D, F, H, J, and L) or one‐way ANOVA (N). All values are means ± SEM. The corresponding *n* values (number of mice) are shown within each sub‐plot.

To analyze the effect of TOP2B knockdown in different tissue/organs, we evaluated the morphology of the kidneys, lungs, livers, hearts, skins, and skeletal muscles of gTOP2B mice using hematoxylin–eosin (HE) staining. Compared to the gLacZ group (23 months old) mice, gTOP2B (23 months old) mice exhibited alleviation of glomerular atrophy in kidney (Figure 3A,B), where glomerular atrophy refers to the shrinkage or loss of function of the glomeruli, the structures responsible for filtration. This condition is a significant marker of kidney aging, reflecting the gradual decline in renal function over time. The presence of glomerular atrophy indicates irreversible kidney damage and is often associated with age‐related renal degeneration (Wang, Yu, et al. 2024). Additionally, gTOP2B mice displayed more regular hepatic lobule structure and reduced anisokaryosis in liver (Figure 3E,F), and a decrease in the average thickness of alveolar septa in lung (Figure 3G,H). Additionally, in gTOP2B mice, there was an enlargement of the cross‐sectional area of skeletal muscle fibers (Figure 3C,D), an increase in the number of nuclei in the left ventricular wall (Figure 3K,L), and thickening of the dermis and epidermis layers and increased number of hair follicles in skin (Figure 3I,J).

Furthermore, we performed SA‐β‐Gal staining and identified SA‐β‐Gal positive cells in kidneys, lungs, livers, hearts, skins, and cortex of 2‐month‐old wild‐type mice, 10‐month‐old wild‐type mice, 23‐month‐old gLacZ, and 23‐month‐old gTOP2B mice (Figure 3M,N). The analysis revealed an age‐dependent increase in the percentage of SA‐β‐Gal positive cells. Administration of gTOP2B treatment via tail vein injection resulted in a significant reduction in the percent of SA‐β‐Gal positive cells in all these tissues compared to the gLacZ mice (Figure 3M,N). These data indicate that TOP2B knockdown effectively reduces senescent cells and alleviates age‐associated characteristics in all the tissues we analyzed. It is worth noting that, although the gTOP2B AAV injection via the tail vein did not decrease the TOP2B protein level in the heart and cortex (Figure 1I,J), the percent of SA‐β‐Gal positive cells still declined in these two tissues relative to the control, suggesting that targeting TOP2B in some of the tissues can produce a systemic effect that is communicated to other tissues.

### 2.4 TOP2B Knockdown Alleviates Various Cellular Aging Hallmarks

**FIGURE 4:**
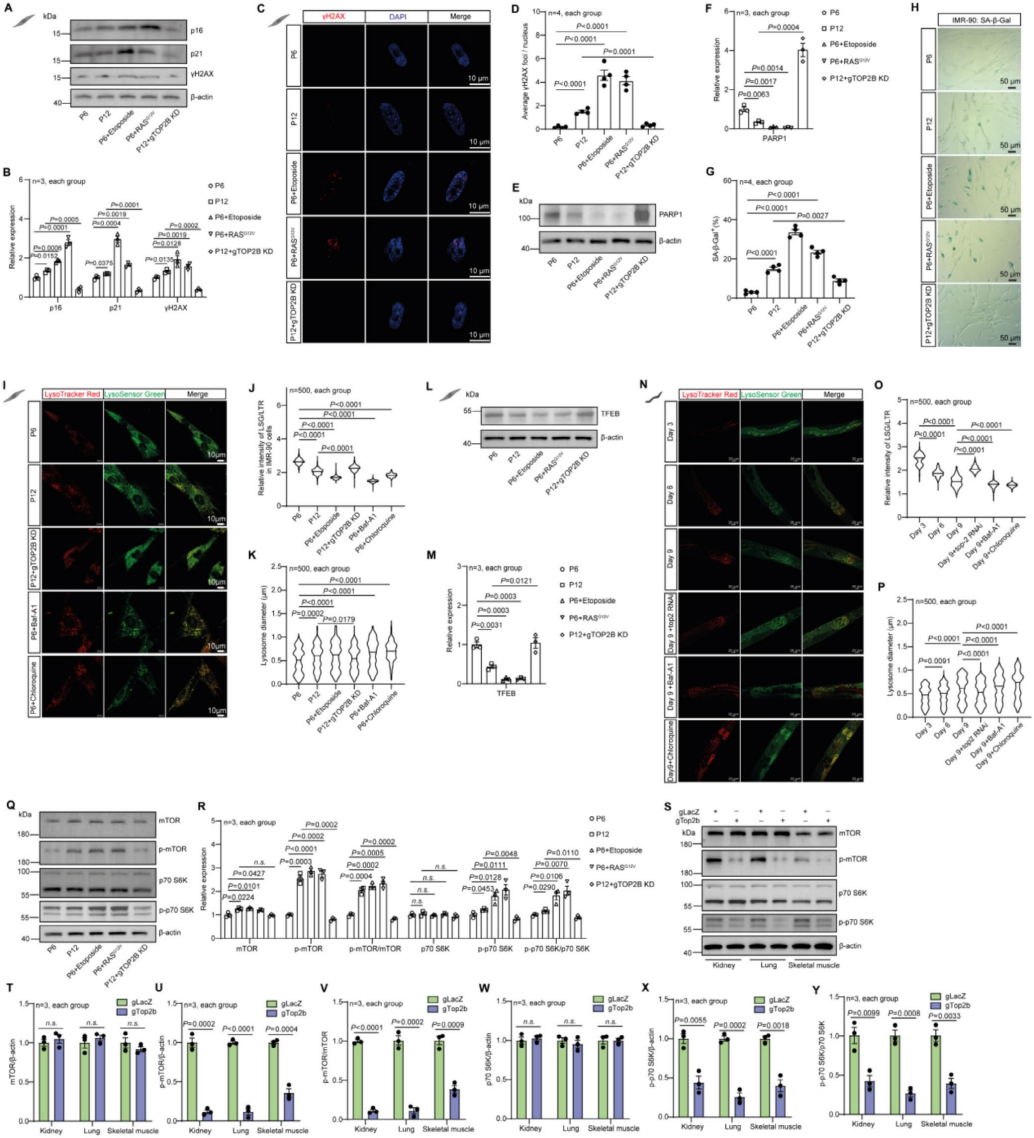
TOP2B knock down reduces various cellular aging hallmarks in human IMR‐90 cells and in various mouse tissues. (A–H) IMR‐90 cells were induced to undergo replicative, stress‐induced, or oncogene‐induced senescence. The protein levels of p16, p21, and γH2AX were detected by western blotting (A, B). γH2AX foci in DAPI‐stained nuclei of IMR‐90 cells were measured by immunofluorescent staining (C, D). The protein level of PARP1 was detected by western blotting (E, F). SA‐β‐Gal staining (blue‐stained cells) and quantification of percent of SA‐β‐Gal positive cells were shown in G, H. (I–P) Confocal fluorescence images of IMR‐90 cells (I–K) and *C. elegans* (N–P) stained by LysoTracker Red DND‐99 (LTR) and LysoSensorTM Green DND‐189 (LSG), and the fluorescence intensity ratio of LSG/LTR was measured as an indicator of lysosomal acidity. (L, M) IMR‐90 cells were induced to undergo replicative, stress‐induced, or oncogene‐induced senescence. The protein level of TFEB was detected by western blotting. The relative intensity of LSG/LTR and average lysosome diameter in IMR‐90 cells (J, K) and *C. elegans* (O, P) were quantified. (Q, R) Western blot analysis of nutrient‐sensing mTOR signaling proteins in IMR‐90 cells. (S–Y) Western blot analysis of nutrient‐sensing mTOR signaling proteins in mouse kidney, lung, and skeletal muscle. Statistical analysis was performed using GraphPad Prism v8.0 software (https://www.graphpad.com). Data were considered statistically significant at *p* < 0.05 calculated by using Student's *t*‐test (T–Y) or one‐way ANOVA (B, D, F, G, M, R) or Kruskal–Wallis test (J, K, O, P). All values are means ± SEM. The corresponding number of samples for IMR‐90 cells, number of worms, and number of mice are shown within each sub‐plot.

To examine the effect of TOP2B knowdown at the cellular/molecular level, we analyzed various aging hallmarks. A prominent hallmark of aging is cellular senescence—the permanent proliferation arrest mediated by activation of cyclin‐dependent kinase inhibitors (CKIs) CDKN1A/p21 and CDKN2A/p16 (Afifi et al. 2023). We thus analyzed the protein expression of p21 and p16 in replicative‐, stress‐ or oncogene‐induced‐senescence of human IMR‐90 cells. Compared with young cells (the 6th generation), p16 and p21 are upregulated in older cells (the 12th generation) and by stress‐ or oncogene‐induced‐senescence (Figure 4A,B). Etoposide‐induced senescence exhibited a stronger p21 increase, whereas oncogenic K‐RAS^G12V^‐induced senescence showed a more significant p16 increase, indicating a distinctive response to different senescence‐inducing signals. We observed that both p16 and p21 were attenuated to a much lower level when TOP2B was knocked down (Figure 4A,B).

DNA damage accumulation is another cellular aging hallmark. DNA double‐strand breaks (DSBs) are potent inducer of cellular senescence, triggering a cascade of DNA damage response pathways that lead to irreversible growth arrest and the acquisition of senescent phenotypes (Yousefzadeh et al. 2021). We found that the number of serine‐139‐phosphorylated H2AX (γH2AX) foci, a marker of DSB, significantly increased in replicative‐senescent, oncogenic K‐RAS^G12V^, and etoposide‐induced senescent IMR‐90 cells and was markedly attenuated after TOP2B knockdown (Figure 4C,D). The total amount of γH2AX as measured by western blotting showed the same trend (Figure 4A,B). Poly(ADP‐ribose) polymerase 1 (PARP1) is the central enzyme for Poly‐ADP‐ribosyl production which is protective to cells during DNA damage (Alemasova and Lavrik 2019). We found that PARP1 protein decreased in replicative‐senescent, oncogenic K‐RAS^G12V^, and etoposide‐induced senescent IMR‐90 cells, but significantly increased after TOP2B knockdown (Figure 4E,F).

We observed increased expression of SA‐β‐Gal activity in replicative‐senescent (P12), oncogenic K‐RAS^G12V^, and etoposide‐induced senescent P6 IMR‐90 cells relative to the P6 IMR‐90 control cells. TOP2B knockdown in P12 cells significantly reduces SA‐β‐Gal compared to P12 control cells (Figure 4G,H). SA‐β‐Gal is generally linked to the increased content of lysosomes (Gorgoulis et al. 2019) and the concomitant decline of lysosomal function correlates with cellular senescence. Lysosomal size increases with aging, reflecting impaired function and the accumulation of cellular waste, which contributes to age‐related diseases (Sun et al. 2020). To analyze whether knockdown of TOP2B can improve aging‐related lysosome dysfunction, we examined lysosome diameter and function in replicative‐senescent and TOP2B knockdown IMR‐90 cells by co‐staining with LysoTracker Red DND‐99 (LTR) and LysoSensorTM Green DND‐189 (LSG), and using Chloroquine (a lysosome inhibitor) and Baf‐A1 (a V‐ATPase inhibitor) treatments as positive controls. The fluorescence intensity ratio of LSG/LTR was measured as a positive indicator of lysosomal acidity (Sun et al. 2020). This measurement quantifies the acidic environment within the lysosome, which is crucial for lysosomal function and its role in cellular homeostasis. Replicative aging, as well as Chloroquine or Baf‐A1 intervention, resulted in an increase in lysosome diameter and a decrease in LSG/LTR ratio, whereas TOP2B knockdown in P12 cells led to a decrease of lysosome diameter and an increase of LSG/LTR ratio relative to the P12 control cells (Figure 4I–K). TFEB is a master regulator of lysosome biogenesis and overexpression of TFEB can induce lysosomal exocytosis (Wang, Martini‐Stoica, et al. 2024). Inhibition of mTORC1 can reduce phosphorylation of several serine residues of TFEB permitting its nuclear translocation and transcriptional function (Cui et al. 2023). We found that in replicative‐senescent, oncogenic K‐RAS^G12V^, and etoposide‐induced senescent IMR‐90 cells, TFEB protein was decreased. TOP2B knockdown significantly increased TFEB level in P12 cells (Figure 4L,M), further indicating improved lysosome function with Top2 reduction.

To examine whether the effect of TOP2B knockdown on lysosome is conserved, we analyzed the effect of Top2 RNAi on lysosome diameter and function in *C. elegans* . The aging process (worms of 3, 6, and 9 days) and Chloroquine or Baf‐A1 treatments led to an increase in lysosome diameter and a decrease in LSG/LTR ratio, while Top2 RNAi had an opposite effect, ameliorating aging‐related lysosome dysfunction (Figure 4N–P).

In summary, the above results indicate that, as an important aging hallmark, lysosomal function decreases with age, including an increase in diameter and a decrease in acidity, while the knockdown of TOP2B or Top2 can significantly reverse these trends both in human IMR‐90 cells and in worms.

Deregulated nutrient sensing is another major hallmark of aging. Aged cells exhibit a deregulated capacity to sense nutrients and poor metabolic status, while effective nutrient sensing contributes to delayed senescence (Xu et al. 2023). Given the crucial role of mTORC signaling in longevity‐related metabolism regulation and nutrient‐sensing, we quantified the protein level of p‐mTOR and mTOR, and its downstream indicator P70‐S6K in IMR‐90 cells (Xu et al. 2023). We found that p‐mTOR/mTOR and p‐P70‐S6K/P70‐S6K ratios increased in replicative‐senescent, oncogenic K‐RAS^G12V^, and etoposide‐induced senescent IMR‐90 cells relative to the P6 control cells, while TOP2B knockdown in P12 cells significantly reduced the two ratios back to those in P6 cells (Figure 4Q,R), indicating that TOP2B knockdown significantly reduces mTORC signaling.

We further tested whether TOP2B knockdown reduces mTORC signaling in vivo in mice. TOP2B knockdown mice exhibited significantly decreased p‐mTOR/mTOR ratio and p‐P70‐S6K/P70‐S6K ratio in the kidney, lung, and skeletal muscle of gTOP2B mice compared to the gLacZ mice, although the mTOR level and the P70‐S6K level themselves did not change, indicating decreased mTORC signaling (Figure 4S–Y).

Together, our data suggest that TOP2B knockdown significantly reduces mTORC pathway signaling both in human cells and in mice. It is known that decreasing mTORC signaling through genetic manipulations or through drug inhibition (such as Rapamycin) is an effective life span‐extending intervention.

### 2.5 Top2 DownRegulation Contributes to Global Transcriptional Changes Indicative of Longevity Promotion

**FIGURE 5:**
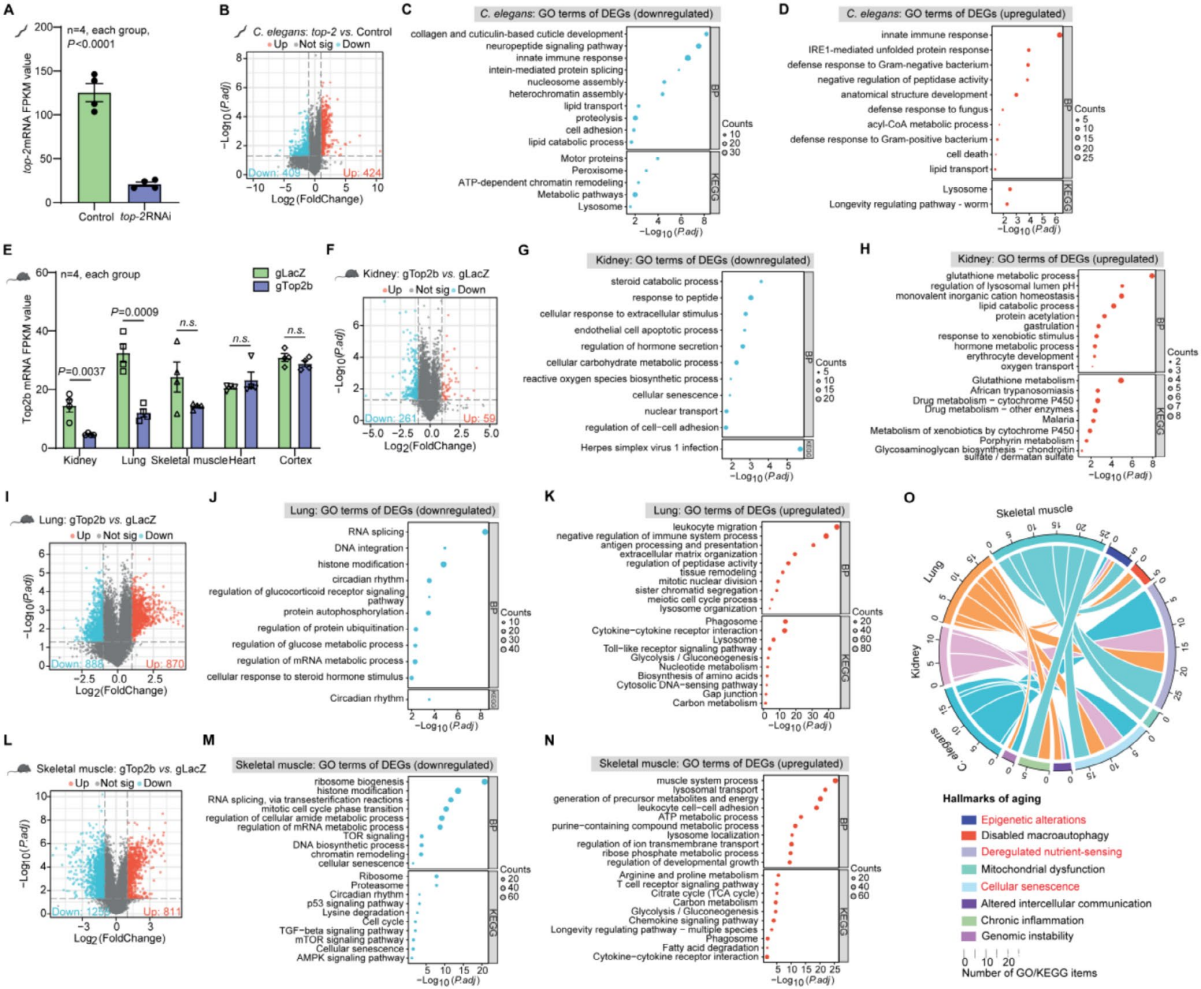
TOP2B knockdown led to changes of the global transcriptional program targeting multiple aging hallmarks. (A) The adjusted Top2 FPKM levels between the control and *top‐2* RNAi‐treated *C. elegans* . FPKM, fragments per kilobase of exon per million mapped fragments. (B) Transcriptome analysis of the upregulated (up) and downregulated (down) DEGs between control and *top‐2* RNAi‐treated *C. elegans* . (C, D) GO analysis identified up (C) and down (D) regulated functional categories in DEGs between control and *top‐2* RNAi‐treated *C. elegans* . BP, biological process; KEGG, Kyoto Encyclopedia of Genes and Genomes. (E) The adjusted TOP2B FPKM levels in the kidney, lung, and skeletal muscle from gLacZ and gTOP2B mice. (F) Transcriptome analysis of the up and down DEGs in the kidney tissues between gLacZ and gTOP2B mice. (G, H) GO analysis identified up (G) and down (H) regulated categories in the kidney tissues between gLacZ and gTOP2B mice. (I) Transcriptome analysis of the up and down DEGs in the lung tissues between gLacZ and gTOP2B mice. (J, K) GO analysis identified up (J) and down (K) regulated categories in the lung tissues between gLacZ and gTOP2B mice. (L) Transcriptome analysis of the up and down DEGs in the skeletal muscle tissues between gLacZ and gTOP2B mice. (M, N) GO analysis identified up (M) and down (N) regulated categories in the skeletal muscle tissues between gLacZ and gTOP2B mice. (O) Circos plot illustrating connections between DEGs due to TOP2b knockdown in different tissues and hallmarks of aging. Statistical analysis was performed using GraphPad Prism v8.0 software (https://www.graphpad.com). Data were considered statistically significant at *p* < 0.05 calculated by using Student's *t*‐test (A and E) or Fisher's exact test and adjusted by the Benjamini–Hochberg method (C, D, G, H, J, K, M, and N). Genes with |log_2_fold change (FC)| > 1 and adjusted *p* value (by the Benjamini–Hochberg method) < 0.05 were considered DEGs (B, F, I, and L). n.s. indicates not significant. All values are means ± SEM. The corresponding *n* values (number of mice) are shown within each sub‐plot.

To explore the potential molecular mechanism underlying the longevity effect of Top2/TOP2B knockdown, we performed RNA‐seq analyses of Top2 RNAi‐treated *C. elegans* (Figure 5A–D; Table S2) and various tissues of TOP2B knockdown mice including kidney, lung, and skeletal muscle (Figure 5E–O, Tables S3–, S5). We observed global changes of transcription profiles in the Top2/TOP2B knockdown mutants compared to the wild‐type control. In Top2 RNAi‐treated *C. elegans* , Top2 mRNA abundance was reduced to 20% of the WT level (Figure 5A), and a total of 424/409 genes were upregulated/downregulated (|Log_2_FC| > 1; *p* value < 0.05, Figure 5B, Table S6). Genes up‐regulated are significantly enriched for several biological processes (BP), including innate immune response and IRE1‐mediated unfolded protein response, indicating increased immune defense and stress resistance. On the other hand, genes downregulated are significantly enriched for collagen and cuticulin‐based cuticle development, indicating a downregulation of developmental/growth‐related process (Figure 5C,D).

CRISPR‐mediated TOP2B knockdown via tail vein injection of AAV virus selectively decreased TOP2B mRNA in the lung, skeletal muscle, and kidney, but not in the heart and cortex, based on the high throughput sequencing data (Figure 5E). This is consistent with the RT‐qPCR and protein expression results (Figure 1H–J). Compared with the gLacZ control mice, gTOP2B mice have 59/261, 870/888, and 811/1255 genes upregulated/downregulated in the kidney, lung, and skeletal muscle respectively (|Log_2_FC| > 1; *p* value < 0.05, Figure 5F,I,L, and Tables S7–, S9).

Functional enrichment analyses indicate that the GO BP (Biological Process) or KEGG terms enriched in differentially expressed genes (DEGs) differ in different tissues. However, the general theme is upregulating processes important for the specific tissue function and downregulating protein synthesis and genes involved in general growth. For example, “glutathione metabolic process” is up‐regulated in kidney, where glutathione plays an important role in antioxidant and detoxification, which is a key function of kidney (Figure 5G,H). In the lung, the most highly enriched categories in the upregulated genes include “leukocyte migration,” “negative regulation of immunce process,” and “anti‐gene processing and presentation,” centered around immune response to infectious agents or irritants. The most significantly downregulated category in lung is “RNA splicing,” a key step in protein synthesis (Figure 5J,K). Skeletal muscle has the biggest change of transcriptome based on the number of DEGs. “Ribosomal genesis” is the most highly enriched in the downregulated genes, indicating downregulation of protein synthesis. Genes responsible for muscle function and energy production are up‐regulated (Figure 5M,N, Extended Data Figure 3, Extended Data Figure 4).

We further compared the DEGs across different mouse tissues. For up‐regulated DEGs, there is almost no overlap among the three tissues (Extended Data Figure 5A). In kidney, a group of genes related to N‐acetyltransferase activity including Nat8, Nat8f5, nat8f6, and nat8f2 were the most significantly upregulated. This is a family of enzymes whose primary role is in the formation of mercapturic acids and in detoxification pathways in liver and kidney (Blum et al. 1990). The most up‐regulated genes in lung were related to immune response. Examples include Acod1, responsible for catalyzing the production of itaconate, an immunoregulatory metabolite, and several cytokines (Cxcl2, Ccl4, Ccl3, and Cxcl10). In skeletal muscle, the most significantly increased genes are related to muscle function. Examples include Actc1, which encodes cardiac muscle alpha‐actin, and Casq1, which serves as the primary calcium‐binding protein in the sarcoplasmic reticulum of skeletal muscle. Interestingly, spermidine biosynthesis‐related genes Amd1 and Smox are highly induced, and spermidine is a known longevity molecule that promotes autophagy (Hofer et al. 2024).

Interestingly, there are 23 shared genes in the downregulated DEGs across tissues (shown in the Venn Diagram, Extended Data Figure 5B,C). Among them are several zinc finger transcription factors whose function are not well characterized, including zfp932, zfp518a, and zfp52. Other genes include Resf1, which positively regulates DNA methylation‐dependent heterochromatin assembly, Dek, which is involved in the regulation of double‐strand break repair via nonhomologous end joining, and Jmjd1c, which has histone H3‐methyl‐lysine‐9 demethylase activity. All these genes are involved in chromatin organization. In addition, two other genes are worth noting. The first one is Arntl, also known as Bmal1, which is a well‐known circadian clock gene. Bmal1 binds E‐box enhancer elements upstream of Period (Per1, Per2, Per3) and Cryptochrome (Cry1, Cry2) genes, capable of activating these genes' transcription. Recently, there has been increasing evidence suggesting the role of biological rhythm in regulating aging (Acosta‐Rodríguez et al. 2021; Wang et al. 2023; Wen et al. 2022). The other gene Apod is a component of high‐density lipoprotein (HDL), whose expression is known to increase with age (Rassart et al. 2020). The rest of the 23 shared genes are generally related to transmembrane transport, stimulus–response, and regulation of protein folding.

To summarize, the functional enrichment analysis and more detailed DEG analysis suggest that most up‐regulated genes are tissue‐specific, aimed at improving the specific tissue function. There are considerable overlaps between downregulated genes, enriched for epigenetic modification and transcriptional regulation. Overall, the global transcriptional changes induced by Top2 knock down are beneficial to longevity promotion by acting on several aging hallmarks, as shown in the Circos plot that links DEGs from different tissues to different aging hallmarks (Figure 5O; Extended Data Figure 5D–G).

### 2.6 TOP2B Knockdown Reprograms the Epigenetic Landscape and Differentially Downregulates Highly Expressed Genes and Genes With Active Promoters

TOP2B plays an essential role in regulating DNA replication and transcription by relieving the torsional stress caused by these processes, and its function in regulating transcription is important for both proliferative and post‐mitotic cells. To further explore the potential upstream mechanism underlying the longevity effect of TOP2B knock down, we analyzed the change of epigenetic landscape and the transcriptional state of DEGs upon TOP2B knock down.

**FIGURE 6:**
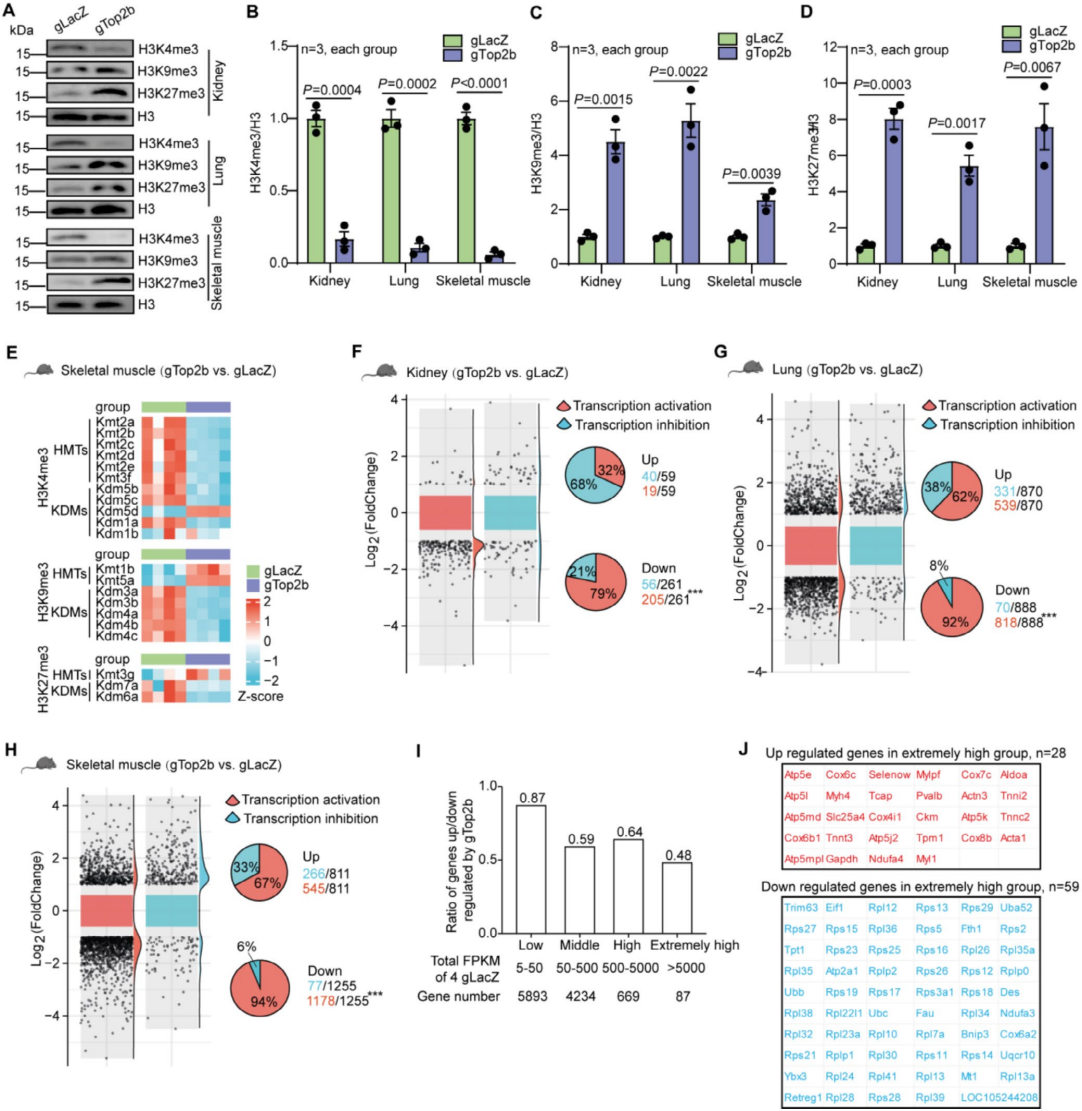
TOP2B reduction reprograms the epigenetic landscape and differentially downregulates genes with active promoters/high abundance. (A–D) Western blot analysis of histone modification markers in mouse kidney, lung, and skeletal muscle. (E) Heatmap depicting the expression levels of genes related to histone methyltransferases (HMTs) and histone lysine demethylases (KDMs) that regulate the trimethylation of histone H3 at lysine residues 4 (H3K4me3), 9 (H3K9me3), and 27 (H3K27me3) in skeletal muscle. (F–H) The transcriptional state of the promoters of DEGs from mouse kidney, lung, and skeletal muscle. The distribution of the log_2_(fold change) of active vs. inactive genes (left panel) and percent of active and inactive genes in the up and down regulated DEGs (right panel) were shown. (I) The ratio of genes up‐regulated/downregulated by TOP2B knockdown in skeletal muscle decreases with the abundance in gLacZ mice. Genes were grouped into low, middle, high, and extremely high abundance groups according to the total FPKM values in gLacZ mice as indicated. (J) Examples of DEGs with extremely high abundance in muscle. Statistical analysis was performed using GraphPad Prism v8.0 software (https://www.graphpad.com). Data were considered statistically significant at *p* < 0.05 calculated by using the Student's *t*‐test (B–D) or Chi‐squared test (F–H). All values are means ± SEM or *n* (%). The corresponding *n* values (number of mice) are shown within each sub‐plot. ***p < 0.001.

We first analyzed various histone markers known to change with age. Compared with the control gLacZ mice, the H3K4me3 (marking active gene promoters and transcription start sites of actively transcribed genes [Xue et al. 2021]) level in TOP2B knockdown mice was significantly downregulated in kidney, lung, and skeletal muscle. In contract, the repressive marks H3K9me3 and H3K27me3 were increased, suggesting that TOP2B knockdown leads to global suppression of transcription (Figure 6A–D).

Interestingly, TOP2B knock down seems to elicit a feedback transcriptional response from epigenetic modifiers‐histone methyltransferases (HMTs) and demethylases (KDMs) that further reinforce transcriptional suppression. For the active H3K4me3 mark, HMTs are significantly downregulated whereas KDMs are upregulated in muscle (Figure 6E). In contract, for the repressive marks H3K9me3 and H3K27me3, the opposite trend is observed.

We next analyzed whether such transcriptional repression is gene‐specific. Numerous studies have indicated that the distribution of TOP2B to target genes is not random (Tiwari et al. 2012; Uusküla‐Reimand et al. 2016). For example, in neurons, TOP2B targets are situated within chromatin regions distinguished by H3K4 methylation, and TOP2B preferentially binds to promoters, with the occupancy positively correlated with an active transcriptional state (Tiwari et al. 2012). We thus hypothesized that in aging mice, TOP2B knock‐down could selectively decrease the expression of those actively transcribed genes, especially those with high abundance.

To address this hypothesis, we utilized the Genome browser (https://genome‐asia.ucsc.edu/) to analyze the transcriptional state of the promoter regions of all the differentially expressed genes (DGEs) in mouse kidney, lung, and skeletal muscle (Tables S10–, S12). In the kidney, the proportion of downregulated DEGs with active transcriptional state in their promoters is 79%, which is significantly higher than the 32% in the up‐regulated DGEs (Figure 6F, Table S10). Similar results were observed in the lung (Figure 6G, Table S11) and skeletal muscle (Figure 6H, Table S12). The distribution of log_2_(fold change) for genes with active transcriptional state shifted significantly toward negative values relative to that of genes with inactive transcriptional state (Figure 6F–H, left panel). These results suggest that TOP2B knockdown preferentially downregulates genes with active transcriptional states whose promoters are enriched for active histone marks.

We further analyzed the selectivity of transcriptional suppression at the gene level using the gene expression data from skeletal muscle, which has the strongest transcriptional response (based on the total number of DEGs) and is relatively homogenous with a smaller number of different cell types. To test whether the transcriptional repression depends on the abundance of gene expression, we group genes into low, middle, high, and extremely high expression groups according to their transcription level in gLacZ mice, and then calculated for each group the ratio between the number of up‐regulated and the number of downregulated genes. We found that although overall there are more downregulated than upregulated genes (the ratio < 1), this ratio decreases with gene abundance, indicating that highly expressed genes are more likely to be downregulated (Figure 6I). Interestingly, for genes in the extremely high group (the total FPKM count > 5000, Figure 6J, Table S5), out of 59 gTOP2B downregulated genes, 41 are ribosomal genes, other 7 are related to ubiquitin and autophagy (Figure 6J). Out of 28 gTOP2B up‐regulated genes, many are involved in muscle development or contraction (Figure 6J). This result from the highly expressed genes is consistent with the global functional annotation of DEGs in skeletal muscle (Figure 4M,N).

## 3 Discussion

Top2 is an essential molecular machine that solves DNA topology problems in the cell. The relief of DNA torsional tension by Top2 is required for DNA replication and transcription (Tammaro et al. 2013; Yan et al. 2016), thus it is crucial for both proliferative cells and post‐mitotic cells. We found that reduction of Top2 or TOP2B (the mammalian homolog) extends lifespan across species, improves the health span of mice and worms, and alleviates age‐related pathologies in various mouse tissues. At the molecular level, TOP2B knock down mitigates many hallmarks of aging, including senescence, DNA damage, and deregulated nutrient sensing. These results suggest Top2 as a promising target for longevity intervention with potentially distinct mechanisms. Our findings also put Top2/TOP2B as a fresh example of genes with antagonist pleiotropy in the context of aging—genes important for development and growth but the downregulation post‐development extends health and life span, similar to the classifical insulin/IGF1 and the mTORC pathways (Kennedy and Lamming 2016; Kenyon 2010).

We would like to note that in *C. elegans* there are three orthologs of Top2: TOP2, CIN4, and R05D3.12. Top2 is the most highly expressed among the three (FPKM count ≈ 125), and is 8 times higher than CIN4. R05D3.12 is very lowly expressed (FPKM count ≈ 4). Top2 RNAi did target both TOP2 and CIN4 (a perfect match to a sub‐sequence of both genes), and we observed a 60% reduction of CIN4 RNA with the 50% RNAi knock down (similar to the 80% reduction of Top2). We suspect that the effect is mainly due to Top2 knockdown, due to its high level of expression, but cannot rule out the possibility that CIN4 knock down also contributes.

We explored the potential mechanisms underlying the pro‐longevity effect of Top2/TOP2B knock down through systematic gene expression profiling of various tissues of mice and analysis of epigenetic markers. We observed that TOP2B reduction changed the epigenetic landscapes of the old mice toward those of the young mice, and differentially downregulated genes with active promoter and high expression. Many of these genes are normally expressed in young tissues. One example is the strong downregulation of ribosomal genes in muscle with TOP2B knockdown, and downregulation of ribosomal gene expression is a major theme of yeast longevity mutants and from worm lifespan RNAi screening (Ganley and Kobayashi 2014; Smith et al. 2008). In addition, there is considerable overlap among downregulated genes in different tissues, including several transcriptional regulators. Intriguingly, such conserved downregulation can trigger a transcriptional cascade that leads to upregulation of tissue‐specific genes that improve tissue‐specific function, such as detoxification in kidney, immune response in lung, and contraction and energy production in muscle. How the conserved downregulation communicates with tissue‐specific transcriptional programs to improve specific tissue function is still unknown and certainly deserves future studies.

Intuitively, TOP2B knock down may exert its pro‐longevity effect by reducing DNA DSBs, as TOP2B is known to co‐localize with DNA DSBs which can be caused by failed re‐ligation. Although we cannot exclude this possibility, our gene expression profiling provided no support for this scenario, with no detectable change of transcriptional signature for DNA damage response and repair pathways.

Our work suggests that TOP2B could be an interesting target for longevity intervention. To develop small molecule drugs targeting TOP2B, two factors need special attention. One is dosage. Our work indicates that there is an optimal level of knock down of Top2 to best extend life span in both yeast and worm (Table S13, Extended Data Figure 6), which is not surprising given the essential function of Top2. More importantly, our work suggests that reducing the level of expression of Top2/TOP2B confers a longevity effect, which may not be achieved by drugs that inhibit the enzyme function at the intermediate steps. A number of topoisomerase inhibitors (such as etoposide, irinotecan, and doxorubicin) have been developed to target cancer; most of these drugs form drug‐Top2‐DNA cleavage complexes, exacerbating DNA double‐strand break (DSB) accumulation (Atwal et al. 2017; Lee et al. 2016; Ma et al. 2021). Previous data from yeast suggest such drugs decrease the lifespan instead of increasing lifespan (Tombline et al. 2017). There is also evidence that inhibition of TOP2B is associated with neurodegenerative disease (Pichierri et al. 2000). Thus it seems that drugs that trap Top2 in the Top2/DNA complex may promote aging (Zhu et al. 2024). Our work suggest that reducing the level of Top2 had pro‐longevity effect, possibly due to its positive effect on modulating transcription and the epigenetic landscape without the negative effect due to increased DNA double‐strand breaks. Thus drugs that specifically target TOP2B for degradation such as PROTACs (Proteolysis Targeting Chimeras) (Liu et al. 2022) could be promising candidates.

## 4 Materials and Methods

### 4.1 Bioinformatic Analysis of Potential Longevity Genes

Yeast replicative lifespan data for knockout strains was taken from (McCormick et al. 2015), with lifespan change percentage and number of cells measured. The gene expression data for knockouts was downloaded from (Kemmeren et al. 2014). We used all the knock‐out strains with both lifespan data and gene expression to perform weighted linear regression for each essential gene, with the gene expression log_2_ foldchange against WT strain as input and lifespan changes (relative to WT) as output, minimizing the weighted cost function for each gene (*j*):

$$\sum_{i=1}^{M}w_{i}{\left(y_{i}-\left(\beta_{0}+\beta_{1j}x_{\mathrm{ij}}\right)\right)}^{2}$$

Where $w_{i}$ is the weight for the strain *i* (calculated from the number of cells used in the lifespan measurement, described below), $y_{i}$ is the lifespan of the strain i, $\beta_{1j}$ is the slope of the regression (slope in the Table S1), $x_{\mathrm{ij}}$ is the gene expression (log_2_ foldchange) of the gene *j* in strain *i*.

Weight assignment:

Since lifespan reliability varies with the number of cells measured, we assigned weights to each lifespan measurement based on the formula:

wi=nini+50

where $n_{i}$ is the number of cells measured in the lifespan experiment. With large $n_{i}$, this weight become close to 1, while for small $n_{i}$ this weight is much smaller, therefore the contribution of the data from such strains will be smaller.

Ranking of the genes:

We then rank the genes by *p* value of the coefficient ($\beta_{1j}$) which quantifies the contribution of the gene to the lifespan prediction.

### 4.2 Mouse Studies Ethics Statement

Mouse experiments were conducted in compliance with International Guidelines and received approval from the Ethical Committee of the University of Electronic Science and Technology of China, Chengdu, China (Ethics Approval No. 106142024050729362).

### 4.3 Lifespan Analysis

Using multiple model organisms such as mice, *C. elegans* , and *S. cerevisiae* , we systematically analyzed the effects of Top2/TOP2B knockdown on the life span. Detailed information is provided in the Supporting Information (Life span analysis).

### 4.4 Histopathological Study

We conducted a comprehensive histopathological study, incorporating SA‐β‐Gal assay, hematoxylin–eosin (HE) staining, quantitative real‐time PCR (qPCR) and Western blotting, with detailed information available in the Supporting Information (Histopathological study).

### 4.5 Phylogenetic Analysis

The identified amino acid sequence of Top2 in FASTA format was uploaded into MEGA 11 software (https://www.megasoftware.net/), involving sequences from 14 different species. The phylogenetic tree was constructed utilizing the Neighbor‐Joining method. Evolutionary distances were computed employing the Poisson correction method, and the nodes of the trees were assessed via bootstrap analysis with 1000 replicates.

### 4.6 Health Span Analysis

We conducted a comprehensive health span analysis utilizing a range of methods, including the FI assessment, rotarod test, open field test, Y‐maze test, elevated zero maze test, novel object recognition test, tail suspension test, body bending frequency and pharyngeal pumping rate of *C. elegans* . Detailed information is provided in the Supporting Information (Behavioral tests).

### 4.7 Construction of Top2 DAmP Strain

The strategy utilized for constructing Top2 DAmP allele followed the previous descriptions (Breslow et al. 2008). In brief, the kanamycin‐resistance (Kan^R^) cassette was inserted immediately downstream of the open reading frame of Top2 via transformation with a PCR product containing the Kan^R^ cassette, which was flanked at both ends by homologous sequences to the targeted locus.

### 4.8 RNAi Experiments

The Top2 and HT115 RNAi clones were generously provided by the Beijing Center for Disease Control and Prevention (Beijing, China). All clones underwent thorough verification via DNA sequencing. For RNAi experiments, synchronized populations of animals were cultivated on OP50‐seeded NGM plates until the late L4 stage or day 1 of adulthood. Subsequently, they were transferred to RNAi plates (NGM supplemented with 100 ng/μL carbenicillin and 1 mM Isopropyl β‐D‐1‐Thiogalactopyranoside) pre‐seeded with bacteria expressing the respective RNAi clone. An empty L4440 vector served as the negative control.

### 4.9 Cell Culture, Senescence Induction, Transfection, Adeno‐Associated Virus (AAV) Production, and AAV Infusion

IMR90 cells were cultured in Dulbecco's modified eagle medium (DMEM) supplemented with 10% fetal bovine serum (FBS), 1% penicillin/streptomycin, glutamine, sodium pyruvate, nonessential amino acids, and sodium bicarbonate. HEK293FT cells were maintained in DMEM with 10% FBS and 1% penicillin/streptomycin. Regular mycoplasma testing was performed using a LookOut mycoplasma PCR detection kit (Sigma). Senescence induced by oncogenic K‐RAS^G12V^ or treatment with etoposide was performed as described.

For transfection of IMR‐90 cells, 1 h prior to transfection, cells were washed with fresh preheated serum‐free DMEM. Then, 1 μg of plasmid was mixed in 50 μL of OptiMEM solution, followed by the addition of 3–5 times the amount of PEI based on DNA quantity. After incubating at room temperature for 15 min, the mixture was added dropwise to IMR‐90 cell culture dishes. The medium was changed after 4–6 h. The gRNA sequences targeting LacZ and TOP2B were designed from the Cas13design website (https://cas13design.nygenome.org/) and sent to Tsingke Biotechnology Co. Ltd. (Beijing, China) for gRNA synthesis. The gRNA sequences are as follows: gTOP2B‐F: aaacTACATCTTCATCATACACCCACA; gTOP2B‐R: cttgTGTGGGTGTATGATGAAGATGTA.

Furthermore, the AAV packaging process for intravenous injection into mouse tail veins was conducted as previously described. The gRNA sequences targeting LacZ and TOP2B were designed from the Cas13design website (https://cas13design.nygenome.org/) and sent to Tsingke Biotechnology Co. Ltd. (Beijing, China) for gRNA synthesis. The gRNA sequences are as follows: gTOP2B‐F: aaacTCATGAATAACTTTGAGAGCCAC; gTOP2B‐R: cttgGTGGCTCTCAAAGTTATTCATGA. Plasmid design involved the selection of the EFS ubiquitous promoter. The AAV‐CasRx‐Triplex‐pregRNA vector was digested with the BSMB1 enzyme and gel purified. The designed gRNAs targeting LacZ and TOP2B were annealed at room temperature for 5 min, then ligated to the vector with T4 ligase at 16°C for 30 min. *Escherichia coli* were transformed to obtain AAV‐CasRx‐Triplex‐TOP2B and AAV‐CasRx‐Triplex‐LacZ plasmids. HEK293FT cells were cultured in DMEM containing 10% FBS; before transfection, the cells were washed with preheated serum‐free DMEM. A mixture of 10 μg pHelper, 5 μg DJ, and 5 μg pAAV in 250 μL OptiMEM solution was combined with 100 μg PEI, incubated at room temperature for 15 min, and then added dropwise to a 10 cm cell culture dish. The medium was changed after 4–6 h. After 72 h, cells were washed with 1× PBS and harvested by centrifugation at 1000 rpm for 3 min. AAV virus was extracted from the cells using the AAVpro Extraction Solution kit (Catalog# 6235, Takara). Fourteen‐month‐old mice (*n* = 30) were injected via tail vein with AAV‐EFS‐CasRx‐Triplex‐TOP2B and AAV‐EFS‐CasRx‐Triplex‐LacZ viruses, with 15 mice per group, each injected with 100 μL of virus at a titer of 1 ×10^12^ vg/mL.

### 4.10 Immunofluorescent Staining

IMR‐90 cells were cultured on autoclaved coverslips and fixed with 4% paraformaldehyde. Following permeabilization with 0.3% Triton X‐100, coverslips were subjected to blocking with a solution containing 10% goat serum and 0.1% Triton X‐100 for 1 h. Primary antibody (γH2AX: Catalog# AF3187; Affinity Biosciences, diluted 1:200) was then applied and incubated overnight at 4°C. The following day, coverslips were washed thrice with TBST before incubating with Goat Anti‐Rabbit IgG H&L (Alexa Fluor 594) (Catalog# ab150080, Abcam, China, diluted 1:1000) for 1 h at room temperature in the dark. Subsequently, coverslips were washed thrice again and immunofluorescence detection was performed using Zeiss LSM800 (Carl Zeiss, Oberkochen, Germany).

### 4.11 LysoSensor Green and LysoTracker Staining

For *C. elegans* , worms were immersed in 100 μL of M9 buffer containing 600 nM LysoTracker Red DND‐99 (LTR, Catalog# 40739ES50, YEASEN, Shanghai, China) and 20 μM LysoSensorTM Green DND‐189 (LSG, Catalog# 40767ES50, YEASEN, Shanghai, China). Staining was conducted for 1 h at 20°C in darkness. Subsequently, worms were transferred to NGM plates seeded with fresh OP50 and allowed to recuperate at 20°C for 1 h in darkness. The fluorescence intensity ratio of LSG to LTR was determined using Zeiss LSM800 (Carl Zeiss, Oberkochen, Germany).

For IMR‐90, cells were cultured on autoclaved coverslips and treated with a solution comprising 200 μL of DMEM containing 10% FBS, 60 nM LysoTracker Red DND‐99 (LTR, Catalog# 40739ES50, YEASEN, Shanghai, China) and 2 μM LysoSensorTM Green DND‐189 (LSG, Catalog# 40767ES50, YEASEN, Shanghai, China). Staining was conducted in darkness for 1 h at 37°C. After three subsequent washes, immunofluorescence detection was carried out using Zeiss LSM800 (Carl Zeiss, Oberkochen, Germany).

### 4.12 Tissue RNA‐Seq Data Processing

Transcriptome sequencing was performed on total RNA extracted from the lungs, kidneys, and skeletal muscles of 23‐month‐old mice (gLacZ group: *n* = 4; gTOP2B group: *n* = 4), as well as from *C. elegans* (control group: *n* = 4; top‐2 RNAi group: *n* = 4) at day 8. RNA‐seq experiments were conducted utilizing a BGISEQ‐500 platform, facilitated by the Beijing Genomic Institution (BGI, China). The sequencing data associated with this study have been deposited in the NCBI Gene Expression Omnibus under the GEO Series accession number GSE278873 (http://www.ncbi.nlm.nih.gov/geo/query/acc.cgi?acc=GSE278873). Detailed information is provided in the Supporting Information (Tissue RNA‐seq data processing).

### 4.13 Statistics

The specific sample sizes are provided in the figure legends. Mean values are depicted with standard error of the mean (s.e.m). Statistical significance was evaluated using the Student's *t*‐test for continuous data and the Chi‐squared test for categorical data. Statistical significance was defined as *p* < 0.05. The locally weighted regression (LOESS) function was used to assess the relationship between the mutant gene's Top2 expression (fold change) and RLS extension in the BY4742 strain. Moreover, with the exception of RNA‐seq data, each experiment was independently replicated at least thrice. Detailed descriptions of the statistical methodologies employed, along with precise *p* values, are elucidated in the respective figure legends. No specific techniques were employed for random sample allocation. All data were inclusively analyzed without exclusions. Data collection and analysis were carried out without blinding to experimental conditions. Analysis was conducted using GraphPad Prism v8.0 software (https://www.graphpad.com).

## Author Contributions

J.Y., H.L., Y.Z., M.Z., and B.X. conceived and designed the experiments. M.Z., M.M., L.L., F.L., L.Y., Y.X., and Z.W. performed the experiments. M.Z., J.Z., Y.P., H.L., and J.Y. performed the bioinformatics analysis. M.Z., H.L., and J.Y. wrote the manuscript with input from all authors. All authors read and approved the final manuscript.

## Conflicts of Interest

The authors declare no conflicts of interest.

## Supporting information


**Table S1.** Correlation between the gene expression changes and the lifespan of the mutants.

**Table S2.** Transcriptome sequencing data of Caenorhabditis elegans.

**Table S3.** Transcriptome sequencing data of kidney.

**Table S4.** Transcriptome sequencing data of lung.

**Table S5.** Transcriptome sequencing data of skeletal muscle.

**Table S6.** Gene Ontology (GO) and Kyoto Encyclopedia of Genes and Genomes (KEGG) analysis of differentially expressed genes (DEGs) in Caenorhabditis elegans.

**Table S7.** Gene Ontology (GO) and Kyoto Encyclopedia of Genes and Genomes (KEGG) analysis of differentially expressed genes (DEGs) in kidney.

**Table S8.** Gene Ontology (GO) and Kyoto Encyclopedia of Genes and Genomes (KEGG) analysis of differentially expressed genes (DEGs) in lung.

**Table S9.** Gene Ontology (GO) and Kyoto Encyclopedia of Genes and Genomes (KEGG) analysis of differentially expressed genes (DEGs) in skeletal muscle.

**Table S10.** Transcriptional state of all differential gene expressions (DGEs) promoter regions in the transcriptomes of kidney.

**Table S11.** Transcriptional state of all differential gene expressions (DGEs) promoter regions in the transcriptomes of lung.

**Table S12.** Transcriptional state of all differential gene expressions (DGEs) promoter regions in the transcriptomes of skeletal muscle.

**Table S13.** Comparative analysis of mutant gene’s top2 expression (fold change) and replicative lifespan extension in BY4742 strain.

**Table S14.** Primer sequences.

Appendix S1.

## Data Availability

The data that supports the findings of this study are available in the Supporting Information of this article.
