# Supplementary material for: Reduction of DNA Topoisomerase Top2 Reprograms the Epigenetic Landscape and Extends Health and Life Span Across Species
Source: Aging Cell. 2025 Feb 12;24(6):e70010. doi: 10.1111/acel.70010 (PMC12151872; doi:10.1111/acel.70010)
Supplement: Supplementary file 15 — Appendix S1. [file ACEL-24-e70010-s007.docx]

**Reduction of DNA Topoisomerase Top2 reprograms the epigenetic landscape and extends health and life span across species**

Man Zhu^1,2^, Meng Ma^2^, Lunan Luo^2^, Feiyang Li^2^, Jiashun Zheng^3^, Yan Pan^2^, Lu Yang^2^, Ying Xiao^2^, Ziyan Wang^2^, Bo Xian^2,#^, Yi Zheng^1,2,#^, Hao Li^3,#^, Jing Yang^1,2,#^

1 Department of Health Management & Institute of Health Management, Sichuan Provincial People's Hospital, University of Electronic Science and Technology of China, Chengdu, China

2 Laboratory of Aging Research, School of Medicine, University of Electronic Science and Technology of China, Chengdu, China
3 Department of Biochemistry and Biophysics, University of California San Francisco, San Francisco, USA
#:Corresponding author: Bo Xian: xianbo@uestc.edu.cn; Hao Li: haoli@genome.ucsf.edu; Yi Zheng: yi_zheng@uestc.edu.cn; Jing Yang: [yangjing1977@uestc.edu.cn](mailto:yangjing1977@uestc.edu.cn)

This PDF file includes:

Supporting text

Extended Data Fig. 1 to Extended Data Fig. 6

Other supporting materials for this manuscript include the following:

Supplementary Table 1 to Table 14

Supporting Information Text

**Life span analysis**

**Mice**

This study included a total of 38 C57BL/6 mice, among which 30 mice (15 males and 15 females, weighing 20-24g each) were randomly and equally assigned to the gLacZ group and the gTop2b group, with genders distributed almost equally between the two groups. These 30 mice, at 8 months of age, were purchased from SIPEIFU (Beijing) Biotechnology Co., Ltd. [Experimental Animal Production License Number: SCXK (Jing) 2019-0010]. Additionally, we purchased 4 mice aged 2 months and 4 mice aged 10 months from SIPEIFU (Beijing) Biotechnology Co., Ltd. Mice underwent daily health assessments to detect any signs of illness. If a mouse displayed severe distress and was not expected to survive beyond 24 hours, as determined by experienced personnel, it was humanely euthanized. Indicators of severe distress included: (1) inability to consume food or water, (2) pronounced lethargy, characterized by minimal response to external stimuli, (3) significant impairment in balance or movement, (4) rapid weight loss over a week or more, or (5) presence of a severely ulcerated or bleeding tumor. The euthanasia age was documented as the most accurate estimate of the mouse's natural life span. Deceased mice were noted during daily inspections, and their bodies were preserved for subsequent examinations. For statistical analysis of survivability, the GraphPad Prism v8.0 software (https://www.graphpad.com) was utilized.

***C. elegans***

The N2 (Bristol) wild-type strain was sourced from the Beijing Center for Disease Control and Prevention (Beijing, China) and maintained at a temperature of 20°C. This strain was cultivated on Nematode Growth Medium (NGM) agar plates supplemented with *E. coli* OP50 bacterial lawn. All experiments were performed using L1 synchronized worms. Wild-type worms raised on *E. coli* OP50 underwent bleaching, followed by L1 synchronization in 1X M9 solution for 16–18 hours at 20 °C. Subsequently, the L1 larvae were pelleted at 2500 rpm for 1 minute, the supernatant was removed, and the larvae were transferred to *E. coli* OP50 plates. Upon reaching the late L4 stage, the worms were transferred to RNAi plates. Life span assessment commenced on the 8th day of adulthood and was conducted every other day thereafter. For all RNAi experiments, an empty L4440 vector was used as the control. Statistical analysis of survivability was performed using GraphPad Prism v8.0 software (https://www.graphpad.com).

***S. Cerevisiae***

The strains utilized in this investigation, namely BY4741 (*MATα his3Δ1 leu2Δ0 met15Δ0 ura3Δ0*) and BY4742 (*MATα his3Δ1 leu2Δ0 lys2Δ0 ura3Δ0*), are of the *S. cerevisiae* genetic background and have been documented previously. Cultivation of yeast strains was executed in YPD complete medium, comprising 2% glucose, 2% peptone, and 1% yeast extract. Growth of yeast strains occurred in selective minimal media at 30°C with agitation set at 250~300 rpm. Analysis of replicative life span (RLS) was conducted through a manual microdissection technique, following established procedures. Pre-culturing of strains took place overnight on YPD plates, with all micromanipulation dissections performed at standard laboratory temperature.

**Health-span analysis**

**Frailty index (FI) assessment**

The FI was evaluated according to established methods. Briefly, each mouse was evaluated for 31 health-related deficits. Each deficit was categorized with a score of 0 (absent), 0.5 (mild), or 1 (severe), as per previous protocols. It's worth noting that for temperature and weight, scores of 0, 0.25, 0.5, 0.75, or 1 were possible, depending on the deviation from reference values in young adult animals, as outlined in prior literature. The cumulative score was then normalized by the total number of deficits assessed, yielding an FI score ranging from 0 to 1.

**Rotarod test**

Prior to experimentation, mice underwent a comprehensive pre-training regimen spanning 5 days to ensure familiarity with the task. Subsequently, they were carefully positioned on a rotating rod, which underwent acceleration from 4 to 40 rpm over 5 minutes. The precise moments at which the mice lost balance and fell from the drum were meticulously recorded, representing their latency to fall. Each mouse underwent a series of five trials conducted separately, with intervals of no less than 5 minutes between each trial. From these trials, both the average latency to fall and the velocity of falling were calculated with meticulous attention to detail. The Rotarod apparatus employed in these experiments was sourced from Jiangsu Sainence Biotechnology Co., Ltd., China (SA102).

**Open field test**

Four gray arenas (measuring 45 × 45 cm) with elevated walls were employed, maintaining a consistent luminosity of 30 lux throughout the habituation and test phases. Mouse cages were acclimated in the behavior room 60 minutes prior to testing. At the commencement of the experiment, mice were situated within the central region of the arena. Each mouse underwent a 5-minute trial within the arena before being returned to its respective home cage. Subsequently, an overhead camera automatically recorded locomotor activity, with subsequent analysis of behavioral patterns conducted utilizing the SMART Video Tracking System V3.0 (Panlab, Harvard Apparatus).

**Y-maze test**

Before commencing the experiment, mouse cages underwent a 60-minute habituation period within the behavior room. Utilizing a gray Y-maze arena measuring 24.6 cm in length and 7.8 cm in width, consistent luminosity at 30 lux was maintained during both training and test phases. For the Y-maze spontaneous alternation test, each mouse was positioned at one extremity of the same arm and allowed 8 minutes of unrestricted exploration. During the Y-maze novel arm test's training phase, one arm was obstructed while the mouse was centrally situated within the maze for 10 minutes of initial exploration. Following this, the mouse was returned to its home cage for a 1-hour interval, and the maze was thoroughly cleaned before subsequent trials. In the test phase, the divider was removed, rendering all three arms accessible. The mouse was then placed at the center and granted 5 minutes to navigate the maze freely. Throughout the experiment, the SMART Video Tracking System V3.0 (Panlab, Harvard Apparatus) recorded both the movement trajectories and duration spent within each arm.

**Elevated zero maze test**

The elevated zero maze comprises a circular platform measuring 600 mm in diameter, partitioned into two segments enclosed by walls standing 6 inches high (designated as the 'shield zone'), and two segments without walls (designated as the 'open zone'). Mice were introduced at the perimeter of the shielded area, with their movements recorded via an overhead camcorder and subsequently analyzed using the SMART Video Tracking System V3.0 (Panlab, Harvard Apparatus). Each trial lasted for 5 minutes. The percentage of time spent in the open zone served as an index of exploratory behavior and inversely as an indicator of anxiety.

**Novel object recognition test**

Four gray arenas (45 × 45 cm) with elevated walls were utilized, maintaining a constant luminosity of 30 lux throughout both habituation and testing phases. Prior to testing, mouse cages were introduced to the behavioral room for a one-hour habituation period. Subsequently, mice underwent a three-day habituation process: on the first day, co-housed mice were introduced to a single arena for 10 minutes collectively, followed by individual 5-minute sessions for each mouse. This individual exposure continued for days 2 and 3. On the fourth day, mice were individually placed in an arena containing two identical objects positioned equidistant from the walls and each other, allowing for 5 minutes of exploration before being returned to their home cage. After a two-hour interval, mice were reintroduced to the same arena, with one familiar object replaced by a novel one, again given 5 minutes for exploration. Locomotor activity and behavioral patterns were automatically recorded by a camera situated above the arena, and subsequently analyzed using the SMART Video Tracking System V3.0 (Panlab, Harvard Apparatus).

**Tail suspension test**

Mouse cages underwent a 60-minute habituation period in the behavior room preceding the experiment. Subsequently, one mouse per experimental condition was gently secured to the tail using tape and positioned in the tail suspension test apparatus, allowing the mouse to hang upside down. The tail suspension test was conducted for a duration of 6 minutes, during which immobility was precisely quantified using the SMART Video Tracking System V3.0 (Panlab, Harvard Apparatus).

**Body bending frequency and pharyngeal pumping rate of *C. elegans***

*C. elegans* specimens were transferred onto an NGM medium devoid of OP50 E. coli. Body bends were quantified as deviations in the movement direction of *C. elegans* (along the X axis), correlated with the movement of the posterior bulb of the pharyngeal ball along the Y axis. Following the stabilization of *C. elegans*, body bending frequency, and pharyngeal pumping rate were documented using stereomicroscopy (Ecoline, Motic) and the Capture 2.1 imaging system (Shenzhen Huaxian Optical Instrument Co., Ltd.).

**Histopathological study**

**Total RNA extraction and real-time quantitative PCR (RT-qPCR)**

Total RNA was extracted from *S.cerevisiae*, *C. elegans,* mouse tissues, and IMR-90 cells using TRIzol reagent (Invitrogen), followed by cDNA synthesis using HiScript III RT SuperMix for qPCR (+gDNA wiper; Vazyme). RT-qPCR was performed following the protocols provided by Applied Biosystems, utilizing a 7500 Fast Real-Time PCR System and the 2 × ChamQ Universal SYBR qPCR Master Mix (Vazyme). Experiments were performed at least three times. The primer sequences are shown in **Supplementary Table 14**. Relative mRNA expression levels were normalized against the respective housekeeping genes: actin (*S.cerevisiae*), actin-1 (*C. elegans*), Gapdh (mouse), or ACTIN (IMR-90) using the 2^-ΔΔCT^ method.

**Western blot analysis**

Various whole cell and tissue extracts were obtained with RIPA buffer (Solarbio) supplemented with PMSF and protease inhibitors. Lysates were briefly sonicated and centrifuged at 12000 × g at 4 °C for 20 min. The protein amount was quantified using BCA protein assay (Solarbio) according to the manufacturer’s instructions. A total of 20 µg was separated on 10 or 15% SDS-PAGE gels and then transferred onto a PVDF membrane 20 or 45 μm (Millipore, Inc.). After blocking with 5% Difco Skim Milk (BD Pharmingen) in TBST solution for 1 h, membranes were incubated with specific antibodies overnight at 4 °C. Following antibodies were used: Top2b (1:2000; Catalog# ER65196; HUABIO), Poly (ADP-ribose) polymerase-1 (PARP-1, 1:1000; Catalog# sc-8007; SANTA CRUZ BIOTECHNOLOGY, INC.), TFEB (1:3000; Catalog# 13372-1-AP; Proteintech), mTOR (1:5000; Catalog# ET1608-5; HUABIO), p-mTOR (1:2000; Catalog# HA600094; HUABIO), p70S6K (1:1000; Catalog# HA721354; HUABIO), p-p70S6K (1:1000; Catalog# HA721803; HUABIO), H3K4me3 (1:1000; Catalog# 91264; Proteintech), H3K9me3 (1:5000; Catalog# M1112-3; HUABIO), H3K27me3 (1:1000; Catalog# R26242; ZenBio), H3 (1:5000; Catalog# M1309-1; HUABIO), p16 (1:1000; Catalog# HA721415; HUABIO), p21 (1:1000; Catalog# HA500156; HUABIO), γH2AX (1:1000; Catalog# AF3187; Affinity Biosciences), and β-actin (1:5000; catalog# ET1702-52; HUABIO). After washing, membranes were further incubated for 60 min at room temperature with the secondary antibodies. The signals were detected with Oriscience Supersensitive ECL Kit (Oriscience Biotechnology Co., Ltd). Finally, the membrane was scanned with a Touch Imager System (e-Blot).

**SA-β-Gal assay**

SA-β-Gal activity in IMR-90 cells and frozen tissue sections was assessed following the manufacturer's guidelines (C0602, Beyotime Biotechnology Ltd, Shanghai, China). Analysis and statistical evaluation were conducted using an optical microscope across randomly chosen fields.

**Hematoxylin-eosin (HE) staining**

HE staining was conducted using a commercial kit (BH0001, POWERFUL BIOLOGY, Wuhan). Briefly, tissue samples underwent formalin fixation, paraffin embedding, and slide preparation. Deparaffinization was achieved with xylene treatment. Subsequently, the specimens were stained with eosin and hematoxylin to visualize cellular cytoplasm and nuclei, respectively. Following dehydration, sections were analyzed using the Motic Digital Slice Scanning System and Motic DSAssistant software (EASYSCAN, Motic, Xiamen).

**Tissue RNA-seq data processing**

For gene expression quantification, uniquely mapped reads were normalized to reads per kilobase of exon per million reads mapped, facilitating the calculation of fragments per kilobase of exon per million mapped reads (FPKMs). Differential gene expression analysis identified genes with |log_2_FC| > 1 and an adjusted *P* value (Benjamini–Hochberg method) < 0.05 as differentially expressed genes (DEGs). The heatmap.2 function from the gplots package version 3.0.1 in R was utilized to generate a graphical representation illustrating Z-score values corresponding to individual genes. DEGs were categorized into upregulated and downregulated genes based on log_2_FC values for enrichment analysis. Gene Ontology (GO) and Kyoto Encyclopedia of Genes and Genomes (KEGG) pathway analyses were conducted using the R package clusterProfiler. Statistical significance was determined for GO terms and KEGG pathways with a *P* value < 0.05. *P* values were calculated using Fisher’s exact test and adjusted via the Benjamini–Hochberg method. Furthermore, to assess differences in biological process signaling pathways among experimental groups, Gene Set Enrichment Analysis (GSEA) was employed using the R package clusterProfiler. Enrichment analysis result with a *P* value < 0.05 was depicted using the enrichplot package.


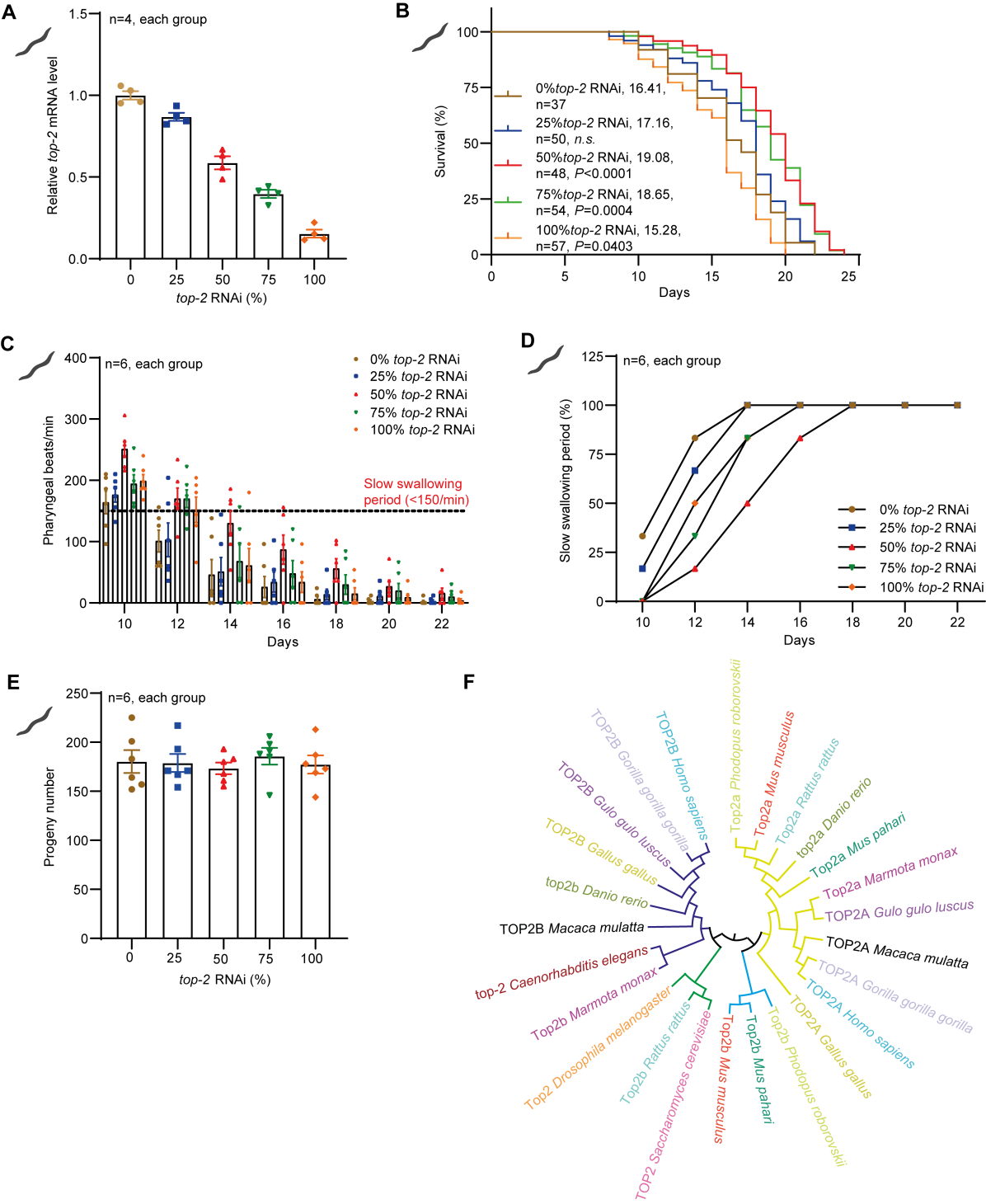


**Extended Data Fig. 1.** Top2 knockdown extended the life span across species.

(A) Gradient Top2 down-regulation was achieved by feeding worms with different proportions of RNAi *E. coli* and wildtype HT115. Relative *top-2* mRNA levels in *C. elegans* for control and *top-2* RNAi groups measured by RT-qPCR.

(B) Life span assay in *C. elegans* for control and *top-2* RNAi groups.

(C-D) Pharyngeal beat assay.

(E) Pregnancy assay.

(F) Phylogenetic analysis of Top2 homologous genes.

A statistical analysis was performed using GraphPad Prism v8.0 software (https://www.graphpad.com). All values are means ± SEM. The corresponding n values are shown within the plots.


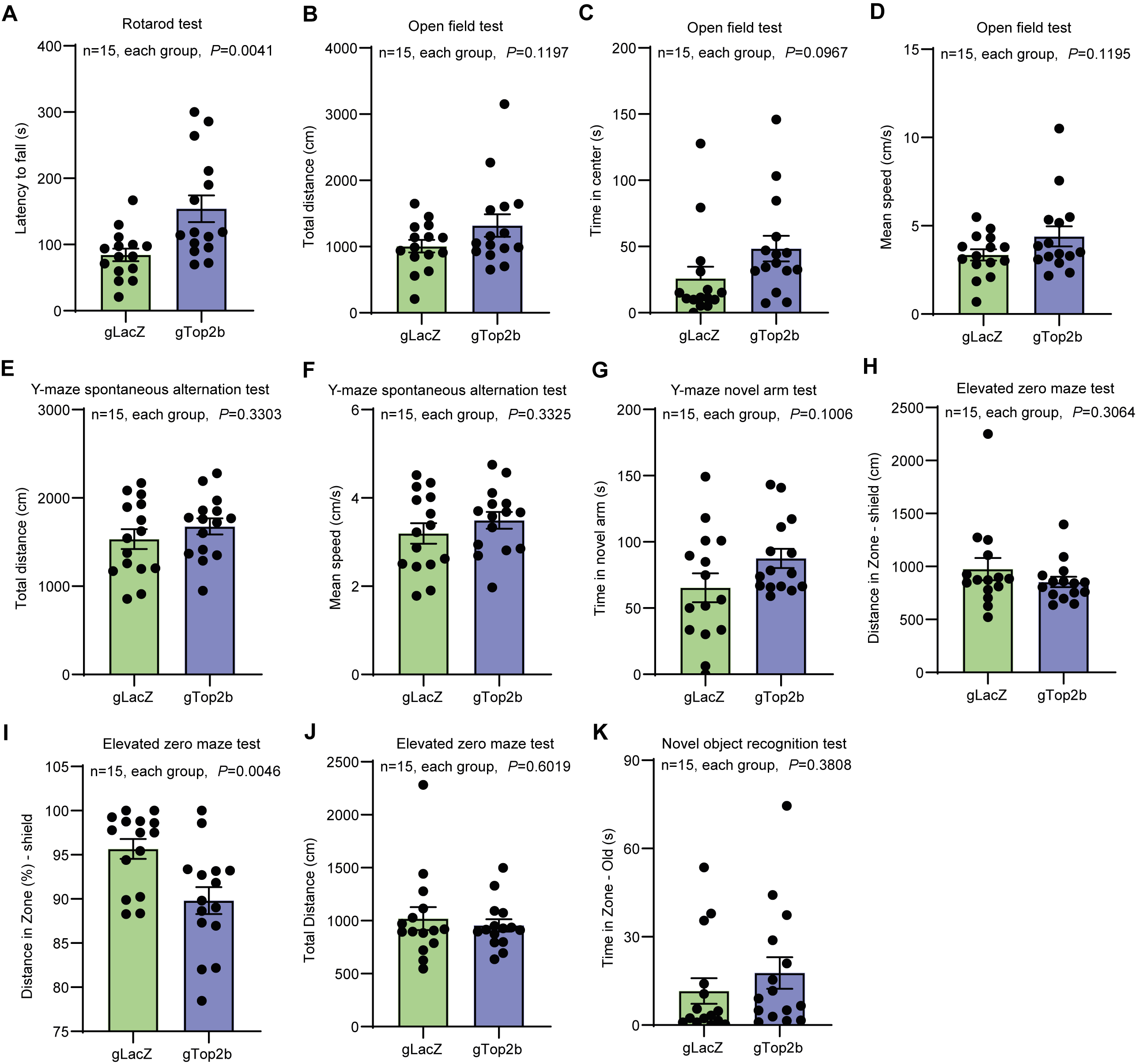


**Extended Data Fig. 2.** Top2b knockdown extended the healthspan in mice.

(A) Rotarod test.

(B-D) Open field test.

(E, F) Y-maze spontaneous alternation test.

(G) Y-maze novel arm test.

(H-J) Elevated zero maze test.

(K) Novel object recognition test.

A statistical analysis was performed using GraphPad Prism v8.0 software (https://www.graphpad.com). Data were considered statistically significant at *P* < 0.05 calculated by using the Student’s t-test. All values are means ± SEM. The corresponding n values are shown within the plots.

**
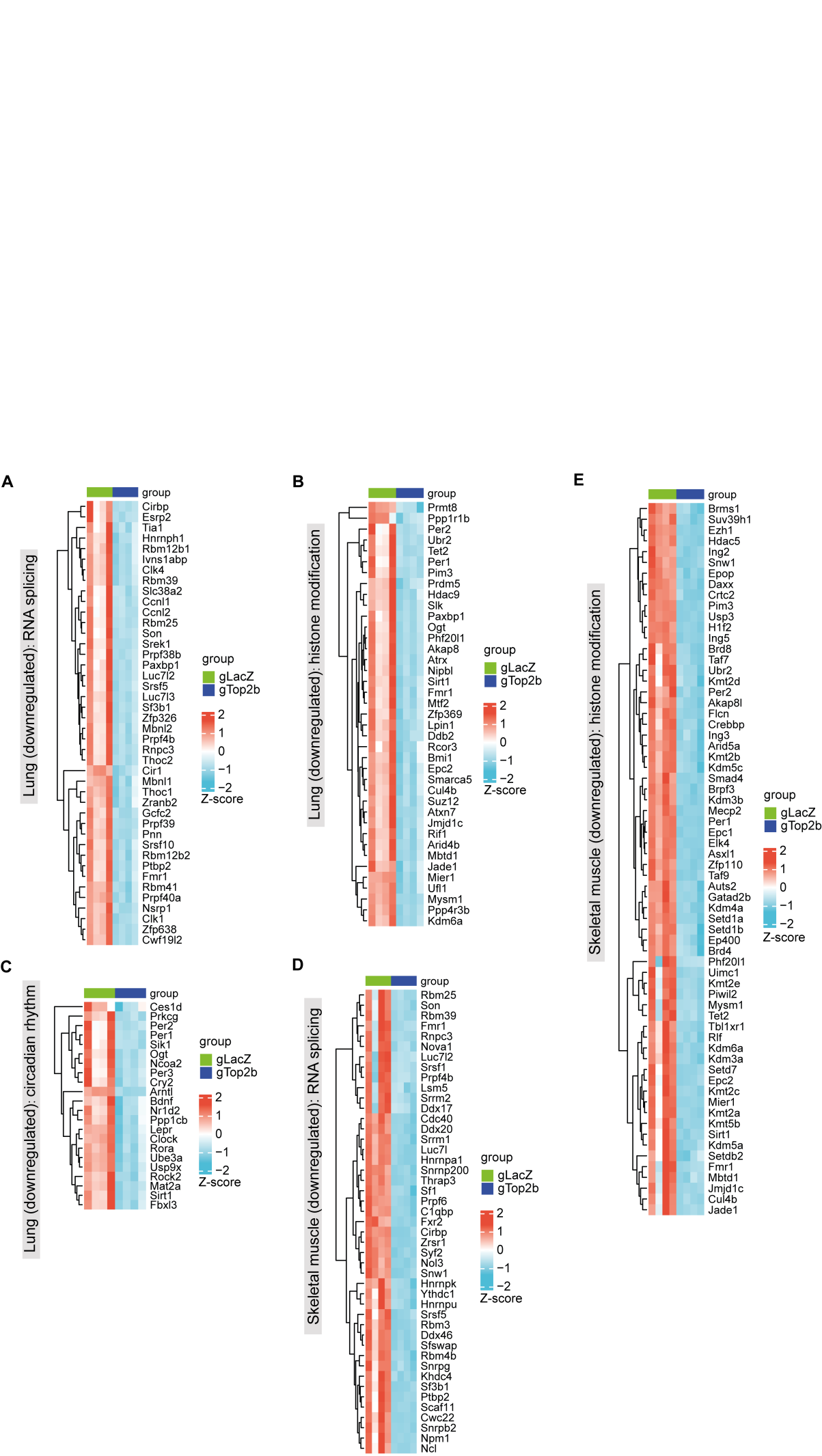
**

**Extended Data Fig. 3.** Top2b down-regulation contributes to global transcriptional changes indicative of longevity promotion.

(A) Heat map showing the DEGs in the ‘RNA splicing’ pathway in lung tissues from gLacZ and gTop2b mice.

(B) Heat map showing the DEGs in the ‘histone modification’ pathway in lung tissues from gLacZ and gTop2b mice.

(C) Heat map showing the DEGs in the ‘circadian rhythm’ pathway in lung tissues from gLacZ and gTop2b mice.

(D) Heat map showing the DEGs in the ‘RNA splicing’ pathway in skeletal muscle tissues from gLacZ and gTop2b mice.

(E) Heat map showing the DEGs in the ‘histone modification’ pathway in skeletal muscle tissues from gLacZ and gTop2b mice.

**
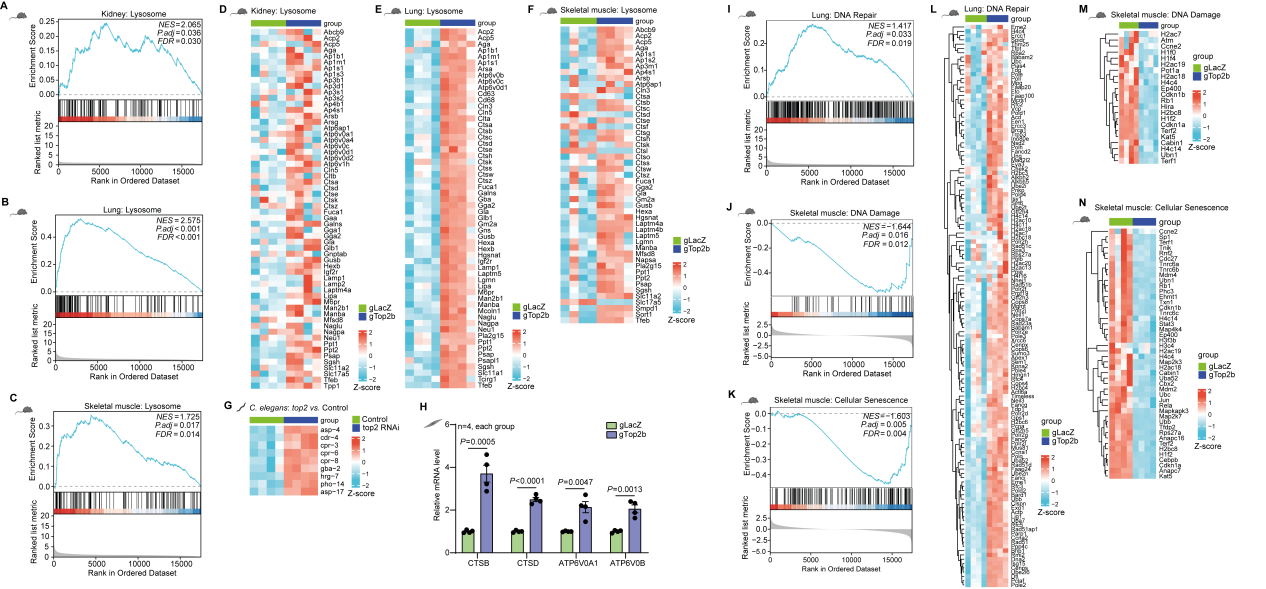
**

**Extended Data Fig. 4.** TOP2 down-regulation contributes to global transcriptional changes.

(A-C) GSEA enrichment curves show the increase of the ‘lysosome’ pathway in the ranked list.

(D-F) Heat map showing the DEGs in the ‘lysosome’ pathway in the kidney, lung, and skeletal muscle tissues from gLacZ and gTop2b mice.

(G) Heat map showing the DEGs in the ‘lysosome’ pathway in the *C. elegans*.

(H) Relative mRNA levels of the ‘lysosome’ pathway-related genes in *IMR-90* for gLacZ and gTOP2B groups measured by RT-qPCR.

(I) GSEA enrichment curve showing the increase of the ‘DNA repair’ pathway in the ranked list.

(J) GSEA enrichment curve showing the decrease of the ‘DNA damage’ pathway in the ranked list.

(K) GSEA enrichment curve showing the decrease of ‘cellular senescence’ pathway in the ranked list.

(L) Heat map showing the DEGs in the ‘DNA repair’ pathway in the lung tissue from gLacZ and gTop2b mice.

(M) Heat map showing the DEGs in the ‘DNA damage’ pathway in the skeletal muscle tissue from gLacZ and gTop2b mice.

(N) Heat map showing the DEGs in the ‘cellular senescence’ pathway in the skeletal muscle tissue from gLacZ and gTop2b mice.


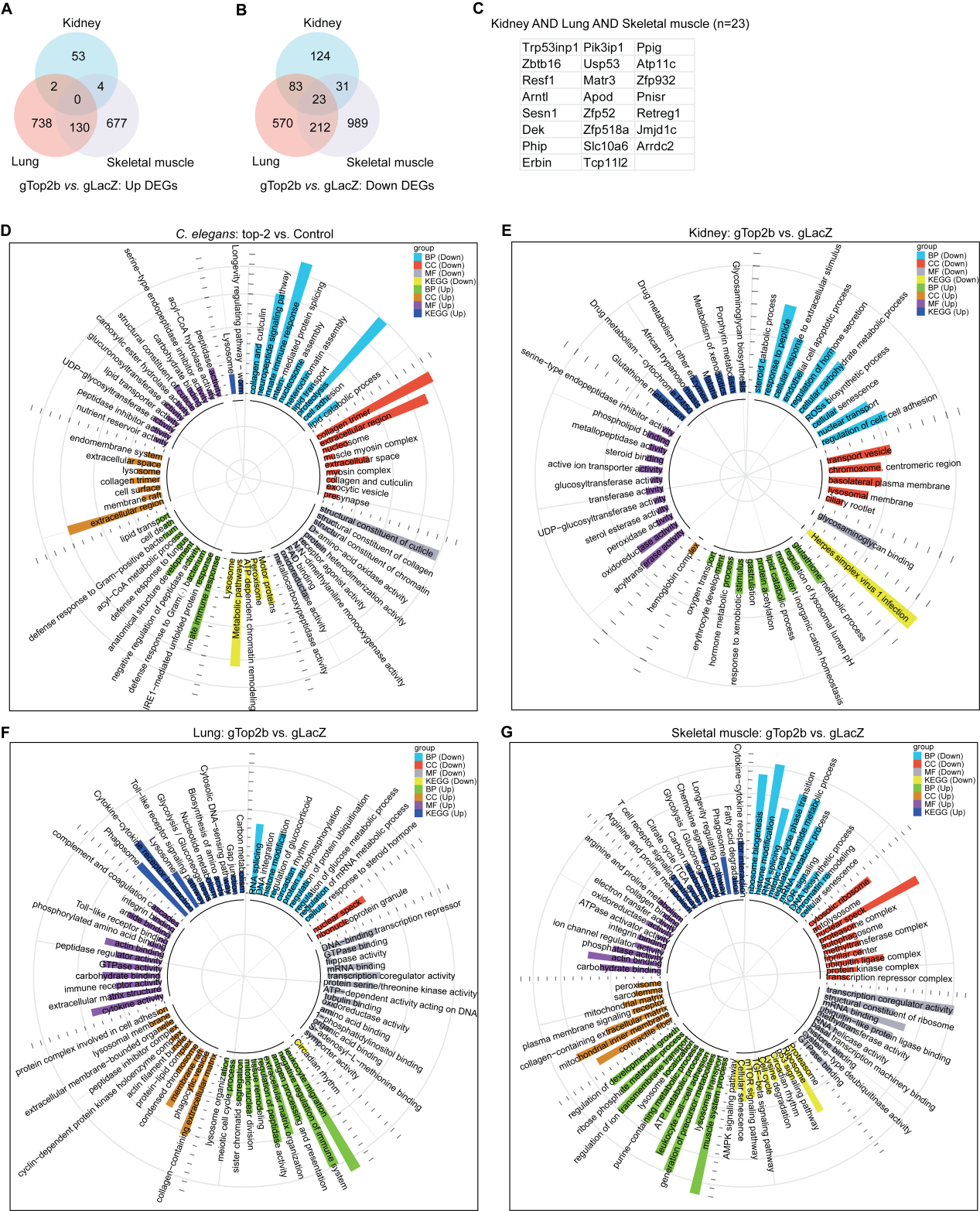


**Extended Data Fig. 5.** TOP2 down-regulation contributes to global transcriptional changes indicative of longevity promotion.

(A) Venn Diagram showing the up-regulated DEGs across mice tissues.

(B) Venn Diagram showing the down-regulated DEGs across mice tissues.

(C) Venn Diagram showing the existence of 23 shared down-regulated-DEGs.

(D-G) Comprehensive GO/KEGG showing the global transcriptional changes after Top2 down-regulation was beneficial to longevity promotion via acting on several aging hallmarks.


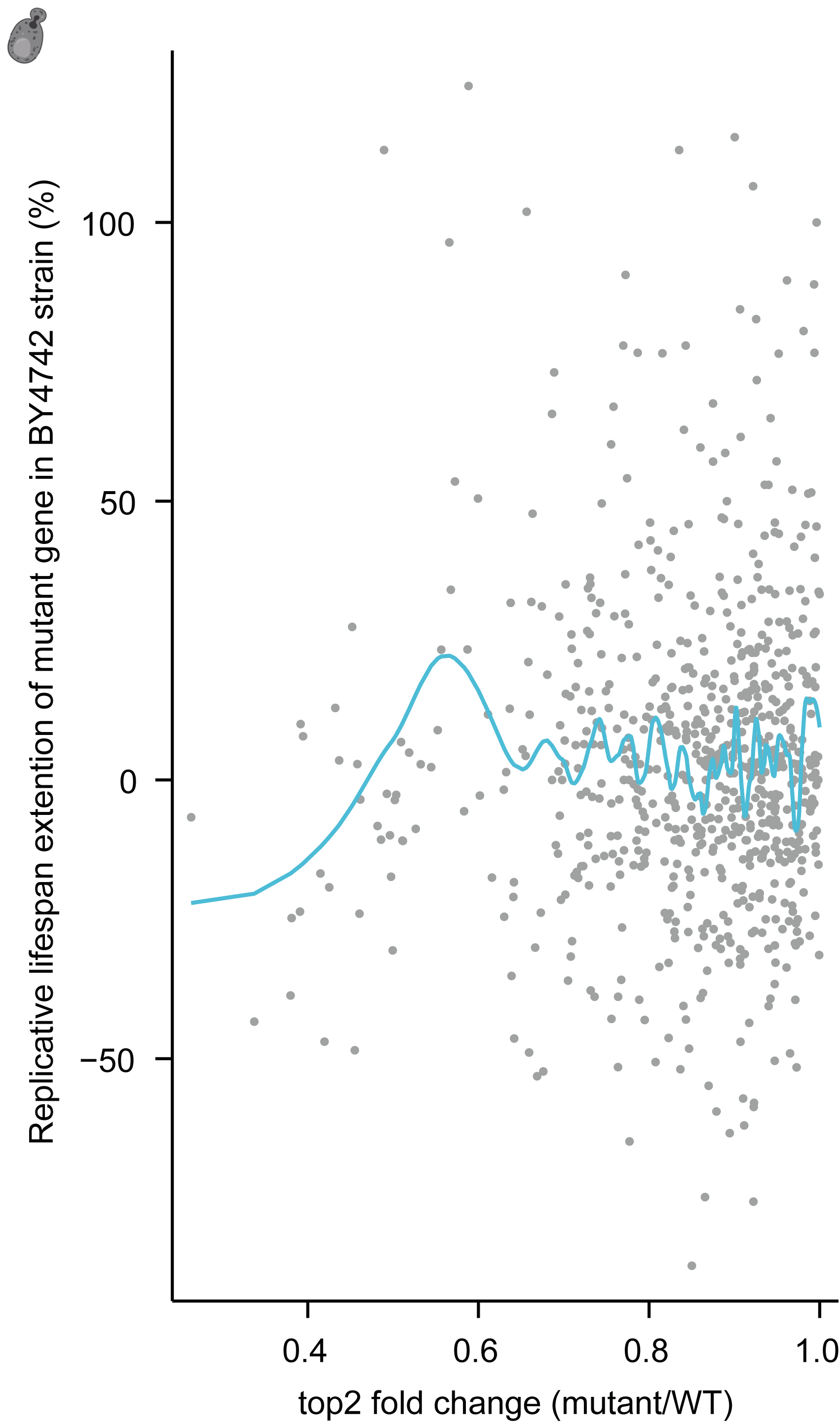


**Extended Data Fig. 6.** Relationship between Top2 variation and replicative life span.

**Supplementary Tables**

**Supplementary Table 1.** Correlation between the gene expression changes and the lifespan of the mutants.

**Supplementary Table 2.** Transcriptome sequencing data of Caenorhabditis elegans.

**Supplementary Table 3.** Transcriptome sequencing data of kidney.

**Supplementary Table 4.** Transcriptome sequencing data of lung.

**Supplementary Table 5.** Transcriptome sequencing data of skeletal muscle.

**Supplementary Table 6.** Gene Ontology (GO) and Kyoto Encyclopedia of Genes and Genomes (KEGG) analysis of differentially expressed genes (DEGs) in Caenorhabditis elegans.

**Supplementary Table 7.** Gene Ontology (GO) and Kyoto Encyclopedia of Genes and Genomes (KEGG) analysis of differentially expressed genes (DEGs) in kidney.

**Supplementary Table 8.** Gene Ontology (GO) and Kyoto Encyclopedia of Genes and Genomes (KEGG) analysis of differentially expressed genes (DEGs) in lung.

**Supplementary Table 9.** Gene Ontology (GO) and Kyoto Encyclopedia of Genes and Genomes (KEGG) analysis of differentially expressed genes (DEGs) in skeletal muscle.

**Supplementary Table 10.** Transcriptional state of all differential gene expressions (DGEs) promoter regions in the transcriptomes of kidney.

**Supplementary Table 11.** Transcriptional state of all differential gene expressions (DGEs) promoter regions in the transcriptomes of lung.

**Supplementary Table 12.** Transcriptional state of all differential gene expressions (DGEs) promoter regions in the transcriptomes of skeletal muscle.

**Supplementary Table 13.** Comparative analysis of mutant gene's top2 expression (fold change) and replicative lifespan extension in BY4742 strain.

**Supplementary Table 14**. Primer sequences.
